# Ghrelin Receptor Deletion or Pharmacological Inhibition Improves Muscle Function in Aging Male Mice

**DOI:** 10.1111/acel.70472

**Published:** 2026-04-15

**Authors:** Haiming L. Kerr, Kora Krumm, Nornubari Myree, Artur Rybachok, Elizabeth Dacek, Brynn Irwin, Siyi Jiang, Lucas Caeiro, Barbara Anderson, Theresa Li, Amanda Chen, Ross Burnside, Jessica Li, Morgan Sydor, David J. Marcinek, Gennifer E. Merrihew, James W. MacDonald, Theo K. Bammler, Michael J. MacCoss, Jose M. Garcia

**Affiliations:** ^1^ Geriatric Research, Education and Clinical Center Veterans Affairs Puget Sound Health Care System Seattle Washington USA; ^2^ Gerontology and Geriatric Medicine University of Washington Department of Medicine Seattle Washington USA; ^3^ Departments of Radiology and Laboratory Medicine and Pathology University of Washington Seattle Washington USA; ^4^ Department of Genome Sciences University of Washington Seattle Washington USA; ^5^ Department of Environmental and Occupational Health Sciences University of Washington Seattle Washington USA

**Keywords:** aging, GHSR‐1a, mice, mitochondria, mitophagy, sarcopenia

## Abstract

Sarcopenia is characterized by age‐related declines in muscle strength and mass, along with impaired physical function. It remains an unmet medical need, and there are no pharmacological interventions approved for this indication. The activation of growth hormone secretagogue receptor (GHSR)‐1a, also known as ghrelin receptor, stimulates food intake and has acute anabolic effects. However, its impact on aging muscles remains uncertain. We examined the effects of GHSR‐1a deletion on sarcopenia measurements (muscle mass, strength, and endurance) by comparing young and aged male GHSR‐1a knockout (KO) and wildtype (WT) mice (6‐, 24‐, and 28‐month‐old). Deletion of GHSR‐1a improved muscle fatigue resistance, endurance, and muscle strength during aging without affecting muscle mass or longevity. Since muscle endurance is closely related to mitochondrial function, we examined mitochondrial biogenesis marker PGC‐1α and mitophagy signaling via *PINK1*/p62 and found them improved in old mice with GHSR deletion. Proteomics analysis also revealed that mitochondrial components remain central for maintaining muscle mass and function. We further investigated the effects of pharmacological inhibition of GHSR‐1a by its inverse agonist, PF‐5190457, in male WT mice. PF‐5190457 mimicked the effects of GHSR‐1a deletion, including improved endurance and increased markers of mitochondrial biogenesis (PGC‐1α) and different mitophagy markers (LC3II and *Bnip3*). PF‐5190457 also reduced body weight and adiposity, which were not observed with GHSR‐1a deletion. Overall, these findings suggest that GHSR‐1a is a promising therapeutic target for age‐related sarcopenia.

## Graphical abstract

Ghrelin receptor (GHSR‐1a) deletion and pharmacological inverse agonism improved muscle function and restored mitophagy and mitochondrial biogenesis markers in aged male mice.

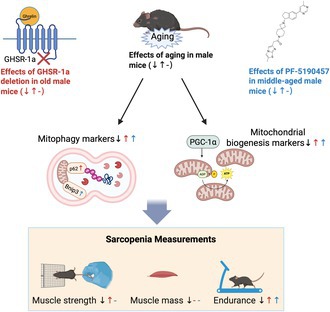

## 1 Introduction

Sarcopenia is an age‐related decline in muscle strength and mass, leading to impaired physical performance (Cruz‐Jentoft et al. 2019), which is an unmet medical need, with no approved pharmacological treatments. So far, exercise is the only recognized treatment for sarcopenia (Izquierdo et al. 2021; Law et al. 2016), while nutritional supplementation (Bauer et al. 2015; Prokopidis et al. 2022) and pharmacological interventions (Becker et al. 2015; Nass et al. 2008; Papanicolaou et al. 2013) have not consistently improved muscle strength and physical function (Anderson et al. 2017). Given that muscle function loss is more pronounced and more clinically relevant than mass loss, and that addressing age‐related loss of muscle function remains a significant challenge, it is essential to investigate potential molecular targets that can improve muscle function. Recently, our group developed a clinically relevant sarcopenia definition in C57BL/6 mice (Kerr et al. 2024). This definition can also help identify the severity of sarcopenia in aged mice based on the most up‐to‐date clinical criteria (Cruz‐Jentoft et al. 2019), emphasizing the importance of muscle strength over muscle mass.

Mitochondria play a pivotal role in regulating muscle function during aging (Borsch et al. 2021; Leduc‐Gaudet et al. 2021; Romanello and Sandri 2015, 2021). Defects in mitochondrial functions accumulate and mitochondrial capacity decreases with aging in animal models and in humans (Borsch et al. 2021; Campbell et al. 2019; Fernando et al. 2023; Kedlian et al. 2024). Impaired coordination between mitochondrial biogenesis and autophagic removal of damaged mitochondria, known as mitophagy, which is a hallmark of sarcopenia, leads to the accumulation of dysfunctional mitochondria, increased oxidative stress, and metabolic inefficiency (Leduc‐Gaudet et al. 2021; Owen and Fry 2024; Romanello and Sandri 2015; Wang, Wen, et al. 2023). Therapeutic strategies that restore mitochondrial quality control, through improvements of mitochondrial biogenesis and mitophagy, hold potential promises for mitigating age‐related muscle function decline in older adults.

Ghrelin increases appetite and growth hormone secretion by activating its receptor, growth hormone secretagogue receptor type 1a (GHSR‐1a) (Kojima et al. 1999). Notably, circulating ghrelin increases slightly with age in mice (Lin et al. 2014; Sun et al. 2007), whereas ghrelin deletion improves muscle function in aged mice and is associated with improved mitochondrial content and metabolism (Agosti et al. 2020; Guillory et al. 2017). As GHSR‐1a is the only known ghrelin receptor, we propose that inhibition of the GHSR‐1a represents a viable anti‐sarcopenia strategy as targeting the receptor may be a more translatable approach than deleting the ghrelin ligand itself. Additionally, we investigated whether pharmacological inhibition of GHSR‐1a with PF‐5190457, an inverse agonist that suppresses the receptor's constitutive activity and reduces ghrelin signaling even in the absence of ligand, recapitulates the effects of GHSR‐1a inhibition, supporting its potential as a therapeutic target. Thus, we hypothesize that GHSR‐1a deletion or treatment with PF‐5190457 improves muscle function via mitochondrial metabolism and alleviates sarcopenia symptoms in mice.

In our study, we demonstrate that GHSR‐1a deletion improves muscle strength and endurance, resulting in fewer cases of severe sarcopenia in aged male mice (24 and 28 months). In aged mice with GHSR‐1a deletion, we also showed increased mitochondrial biogenesis and mitophagy markers in skeletal muscle. Moreover, proteomic analyses revealed that GHSR‐1a deletion altered the correlations between specific pathways and sarcopenic measurements with aging, including immune responses, mitochondrial and amino acid metabolism, and extracellular matrix (ECM) pathways. PF‐5190457 administration for 28 days also improved endurance and enhanced markers of mitochondrial biogenesis and mitophagy in muscles while reducing adiposity. In summary, our findings suggest that GHSR‐1a is a promising therapeutic target for alleviating sarcopenia, offering a viable avenue for pharmacological intervention.

## 2 Results

### 2.1 GHSR‐1a Deletion Improves Muscle Function in Aged Male Mice

**FIGURE 1:**
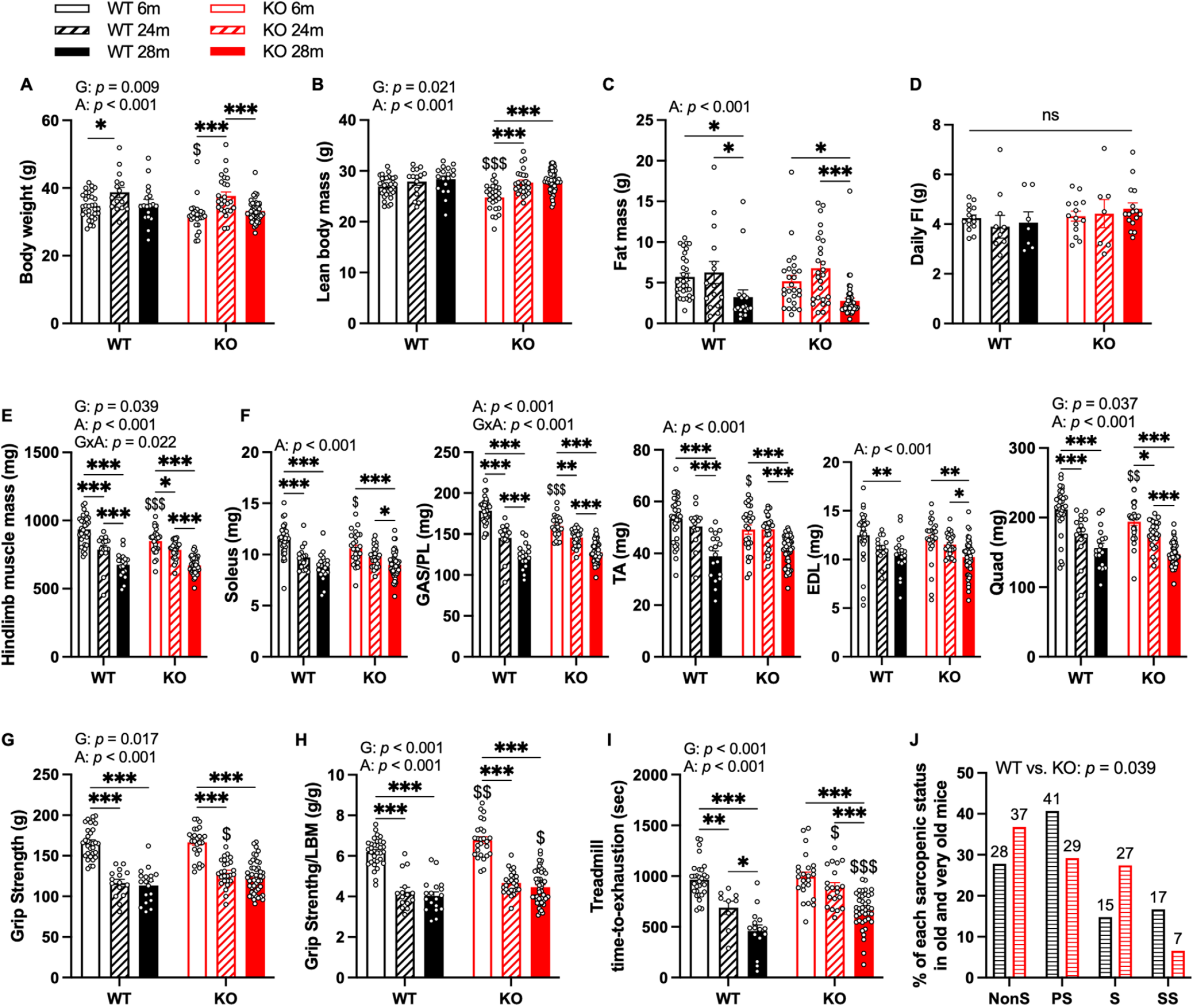
GHSR‐1a deletion improved muscle function without affecting muscle mass in 24‐ and 28‐month‐old male mice. Body composition, muscle mass, and muscle function were evaluated in 6‐months (m), 24 m, and 28 m old male GHSR‐1a wild‐type (WT) and knockout (KO) mice, including (A) body weight (grams, g), (B) lean body mass (LBM, g), (C) fat mass (g), (D) daily food intake (g), (E) total hindlimb muscle mass (mg), (F) individual hindlimb muscle mass (mg), (G) grip strength (g), (H) grip strength normalized to LBM (g/g), (I) treadmill running time (seconds, s, time‐to‐exhaustion), and (J) rate of each sarcopenic status (nonsarcopenic [NonS], probable sarcopenic [PS], sarcopenic [S], or severe sarcopenic [SS]). Two‐way ANOVA was performed to detect age and genotype differences (A–H), followed by Tukey post hoc tests. Significant main effects of genotype (G), age (A), or their interaction (G × A) are presented in each figure. $, $$, $$$: Different from the same‐aged WT mice (*p* < 0.05, 0.01, and 0.001); *, **, ***: Difference between age groups with the same genotype (*p* < 0.05, 0.01, and 0.001). Pearson Chi‐square test was performed to detect genotype differences in the rates of sarcopenic status (J). Data are shown as mean ± SE. Sample sizes: *N* = 32, 15, 18, 26, 26, and 47 for panels A–F; *N* = 30, 9, 15, 24, 20, and 41 for panel I (left to right bars). N for panel J is indicated on top of each bar.

To understand the effects of GHSR‐1a deletion on sarcopenia in aged mice, we evaluated body composition, food intake, treadmill running time, grip strength, and hindlimb muscle mass in male GHSR‐1a WT and KO mice aged 6, 24 and 28 months. In the 6‐month groups, animals with GHSR‐1a deletion (KO) exhibited lower body weight and lean body mass (LBM) compared to WT mice (Figure 1A,B). Both genotypes exhibited increased body weight in 24‐month‐old mice, but not in 28‐month‐old mice, compared to 6‐month‐old mice (Figure 1A). A significant main effect of age was observed in LBM; however, the increase in LBM with age was only significant in KO mice (Figure 1B). As ghrelin may promote water retention via vasopressin release (Ishizaki et al. 2002), we assessed free water content (g) based on body composition analysis in young and very old mice. No significant differences were observed between WT and KO mice despite aging increasing free water content in both genotypes (Figure S1). Furthermore, 28‐month‐old mice displayed lower fat mass compared to 6‐ and 24‐month‐old mice in both genotypes (Figure 1C). No significant difference in daily food intake was found across groups (Figure 1D). Total hindlimb muscle mass was calculated as the combined weight of the bilateral soleus, gastrocnemius/plantaris complex (GAS/PL), tibialis anterior (TA), extensor digitorum longus (EDL), and quadriceps (Quad). It was lower in 6‐month‐old KO mice compared to same‐aged WT mice, it decreased with age in both genotypes, and no genotype difference was found in older groups (Figure 1E). Similarly, individual hindlimb muscles demonstrated age‐related atrophy in both genotypes, and KO mice showed reduced muscle mass at 6 months in soleus, GAS/PL, TA, and Quad (Figure 1F). Despite the absence of genotype differences in muscle mass at older ages, 24‐month‐old KO mice exhibited higher grip strength compared to age‐matched WT mice (Figure 1G). Six‐ and 28‐month‐old KO mice demonstrated 9.4% and 10.6% higher normalized grip strength (grip strength/LBM) compared to WT mice, respectively (Figure 1H); while this measurement declined with age in both genotypes. Twenty‐four‐ and 28‐month‐old KO mice showed 29.5% and 44.3% higher treadmill running times compared to WT mice, respectively, and KO mice also showed a delayed decrease in treadmill running time during aging compared to WT (Figure 1I). Additionally, we assessed sarcopenic status in mice aged 24 months or older based on our previously established definition in C57BL/6 mice, which incorporates measurements of grip strength, muscle mass, and treadmill running time (Kerr et al. 2024). Mice were categorized into four groups according to the number of deficits present across these three parameters (details in Methods): nonsarcopenic (NonS), probable sarcopenic (PS), sarcopenic (S), and severe sarcopenic (SS). Among aged mice, a significant genotype difference was observed between WT and KO, with KO mice exhibiting a higher proportion of NonS and S, and a lower proportion of PS and SS compared to the WT group (Figure 1J).

### 2.2 GHSR‐1a Deletion Alters Fiber Type Composition in 24‐Month‐Old Mice but Has no Effect on Fiber Size

**FIGURE 2:**
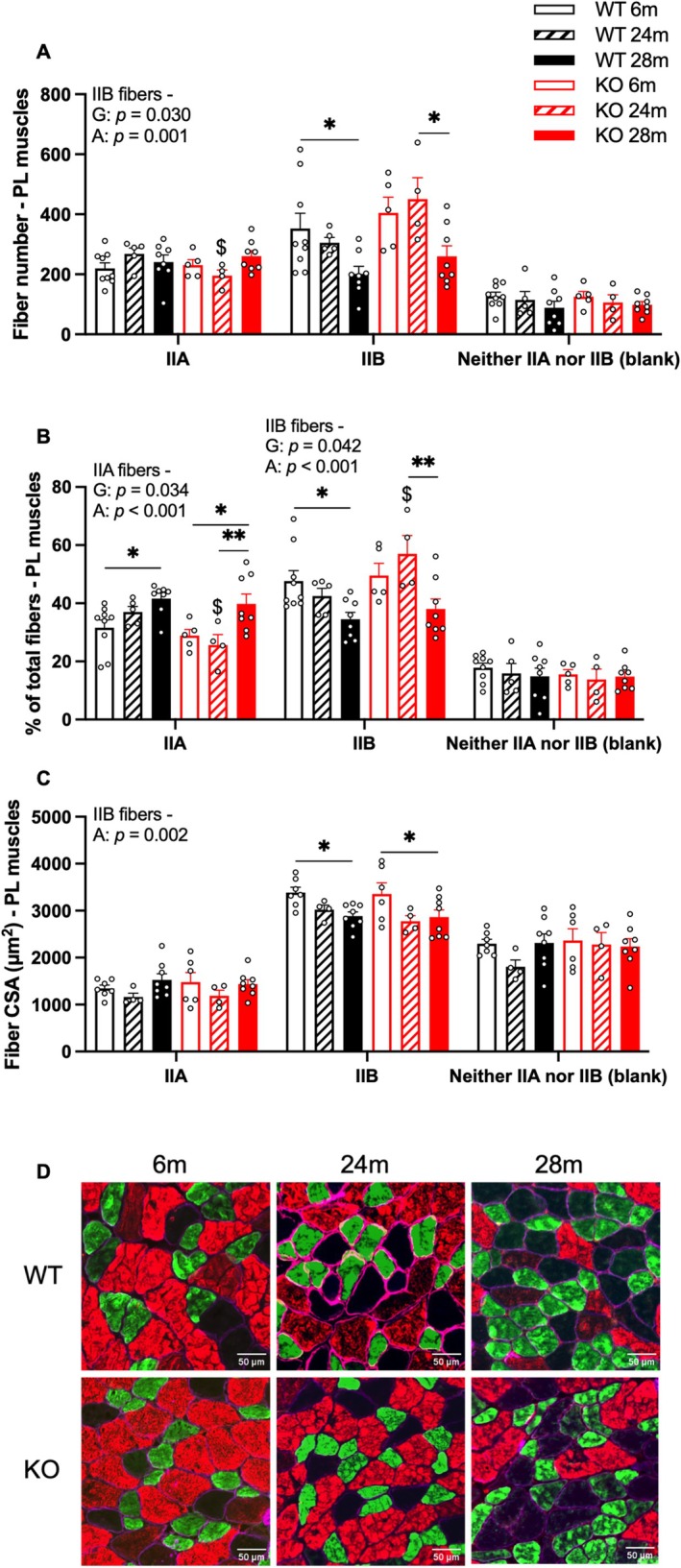
GHSR‐1a deletion altered fiber type composition in 24‐month‐old mice but had no effect on fiber size. Immunohistochemistry of myosin heavy chain (MHC) type IIA, IIB, and neither IIA or IIB (stained as blank) fibers in PL muscles from 6‐months (m), 24 m, and 28 m old male WT and KO mice. (A) Fiber number in PL muscles by MHC type, (B) percentage of each fiber type in PL muscles, and (C) CSA of fibers by MHC type in PL muscles. (D) Representative images of immunohistochemistry MHC staining for IIA (green), IIB (red), and neither IIA nor IIB (blank) in PL, with membranes stained for dystrophin (magenta). Two‐way ANOVA was performed to detect age and genotype differences, followed by Tukey post hoc tests. Significant main effects of genotype (G), age (A), or their interaction (G × A) are presented in each figure. $: Different from the same‐aged WT mice (*p* < 0.05); *, **: Difference between age groups with the same genotype (*p* < 0.05 and 0.01). Data are shown as mean ± SE. Sample sizes: *N* = 9, 5, 8, 5, 4, 8 for panels A–B; *N* = 7, 4, 8, 6, 4, 8 for panel C (left to right bars).

We examined fiber composition and cross‐sectional area (CSA) in plantaris (PL) muscles. In WT mice, aging significantly reduced the number of type IIB fibers, with 28‐month‐old mice exhibiting a lower fiber count compared to 6‐month‐old mice (Figure 2A). KO showed a similar pattern, though IIB fiber number did not decrease until 24 months. Also, KO mice exhibited fewer IIA fibers but more IIB fibers at 24 months (though the latter was not significant, *p* = 0.060; Figure 2A). In terms of the percentage of fibers, WT mice showed an increased proportion of IIA fibers, while a decrease in IIB fibers with aging (28 months vs. 6 months, Figure 2B). KO mice showed similar age‐related changes in proportions of IIA and IIB fibers at 28 months compared to both 6‐ and 24‐month‐old groups, though the difference in IIB fibers between 6‐ and 28‐month‐old KO mice was not statistically significant (*p* = 0.090). In addition, 24‐month‐old KO mice had a higher percentage of IIB fibers and a lower percentage of IIA fibers compared to WT mice (Figure 2B). Type IIB CSA decreased with age in 28‐month‐old mice of both genotypes (Figure 2C).

### 2.3 Muscles From GHSR‐1a KO Mice Are More Fatigue‐Resistant Than Those From WT Mice

**FIGURE 3:**
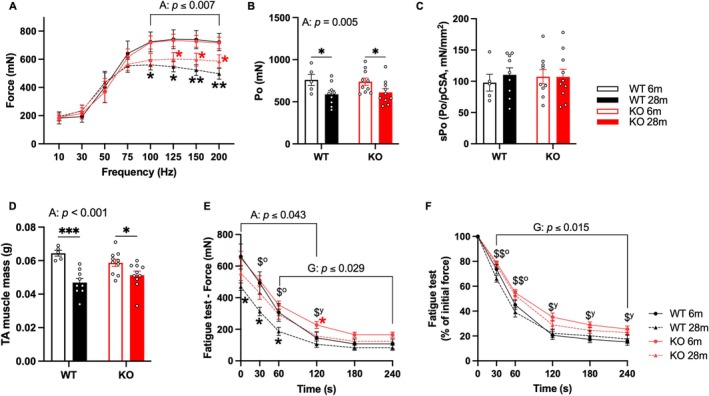
GHSR‐1a KO mice were more fatigue‐resistant than WT mice. Contractile properties of tibialis anterior (TA) muscles from 6‐ and 28‐month (m)‐old male WT and KO mice were assessed. (A) Contractile force (mN) in response to stimulation at various frequencies (Hz). (B) Peak force (Po, mN) recorded as the highest force production during the force‐frequency regimen. (C) Specific force of the TA muscle calculated as peak force divided by physiological cross‐sectional area (mN/mm^2^). (D) TA muscle mass (g). (E) Contractile force (mN) during a fatigue test with muscle stimulation at 150 Hz every 2 s for 4 min. (F) Normalized contractile force during the fatigue test expressed as a percentage of initial force. Two‐way ANOVA was performed to detect age and genotype differences, followed by LSD post hoc tests. Significant main effects of genotype (G), age (A), or their interaction (G × A) are presented in each figure. *, **: Different from the 6 m group with the same genotype (*p* < 0.05 and 0.01, black: WT, red: KO). $^y^: Different from the 6 m‐old WT (*p* < 0.05); $^o^, $$^o^: Different from the 28 m‐old WT (*p* < 0.05 and 0.01). Significant main effects (ME) of age or genotype are indicated in panels D and E. Data are presented as mean ± SE. Sample sizes: *N* = 5, 9, 10, and 10 for WT 6 m, WT 28 m, KO 6 m, and KO 28 m in panels A–D; *N* = 5, 7, 10, and 10 for the same groups in panels E, F.

To understand the effects of GHSR‐1a deletion on muscle contractile function, we performed in situ muscle physiology in tibialis anterior (TA) muscles from 6‐ and 28‐month‐old WT and KO male mice. Force production of TA muscles decreased with aging during the force‐frequency test. In WT mice, the decline began at 100 Hz, whereas in KO mice, it started at 125 Hz (Figure 3A). Both genotypes exhibited age‐related decreases in peak force (Po, Figure 3B), but specific force remained unchanged (Figure 3C). TA muscle mass in the mice used for muscle physiology showed significant age‐related declines in both genotypes, while no genotype difference was detected (Figure 3D). During the fatigue test (150 Hz every 2 s), WT mice experienced age‐related declines in absolute force from 0 to 60 s, while KO mice showed a decline at 120 s. Compared to WT mice, KO mice were less fatigued, demonstrating higher absolute and normalized force at 30 and 60 s in 28‐month‐old mice, and higher absolute force at 120 s and normalized force at 120–240 s in 6‐month‐old mice (Figure 3E,F).

### 2.4 GHSR‐1a Deletion Increases Markers of Mitochondrial Biogenesis and Mitophagy in Skeletal Muscles

**FIGURE 4:**
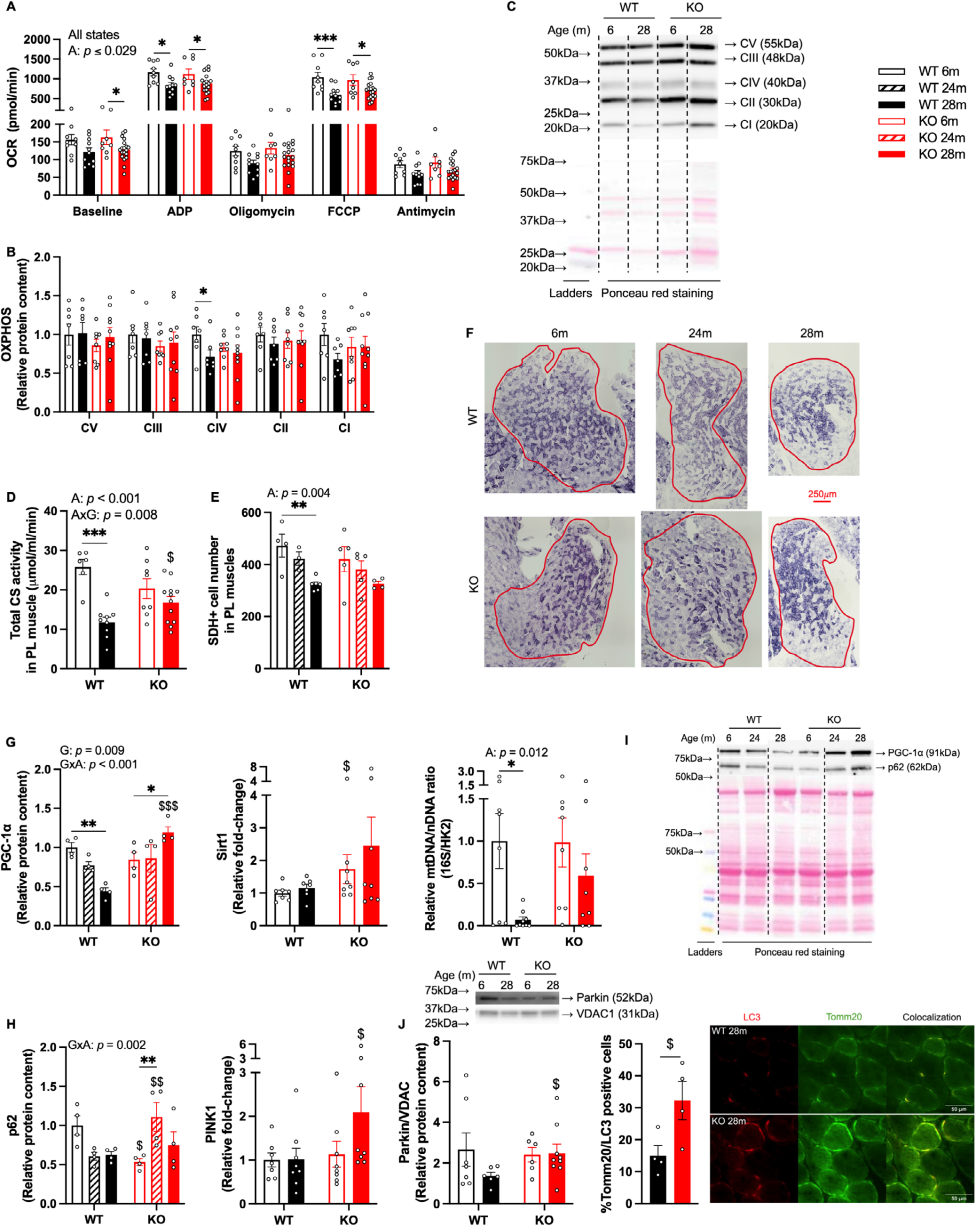
Aging impaired multiple mitochondrial components in WT mice, while GHSR‐1a deletion increased markers of mitochondrial biogenesis and mitophagy in skeletal muscles. (A) Mitochondrial respiration, measured as the oxygen consumption rate (OCR) at different states, including baseline, and after stimulation with ADP, oligomycin, FCCP, and antimycin, in isolated mitochondria from PL muscles (pmol/min, *N* = 9, 11, 8, 19 for the bars from left to right). (B, C) Protein levels of oxidative phosphorylation (OXPHOS) complexes in isolated mitochondria measured using Western blotting (*N* = 7, 7, 8, 10). (C) Representative Western blots and Ponceau red staining for OXPHOS proteins. (D) Citrate synthase activity in total PL muscle (μmol/mL/min, *N* = 6, 9, 8, 12). (E) Percentage of SDH‐positive area in PL muscles and (F) representative images of SDH staining. Red lines contour the PL muscle area (*N* = 4, 3, 6, 5, 5, 4). (G) Mitochondrial markers, including relative protein content of PGC‐1α (*N* = 4), mRNA expression of *Sirt1* (*N* = 7–8), and relative expression of mtDNA (16S, normalized to nuclear DNA HK2, *N* = 8, 9, 8, 8). (H) Mitophagy markers, including relative protein content of p62 (*N* = 4) and mRNA expression of *PINK1* (*N* = 7–8). (I) Representative Western blotting images of PGC‐1α, p62, and Ponceau red staining. (J) Relative protein level of Parkin in isolated mitochondria (normalized to VDAC1. *N* = 7, 6, 6, 9), and percentage of muscle fibers with positive TOMM20 and LC3 colocalization with representative images (*N* = 4). Two‐way ANOVA was performed to detect age and genotype differences, followed by Tukey or LSD post hoc tests. Significant main effects of genotype (G), age (A), or their interaction (G × A) are presented in each figure. $, $$, $$$: Significantly different from the same‐aged WT mice (*p* < 0.05, 0.01, and 0.001); *, **, ***: Significantly different between age groups with the same genotype (*p* < 0.05, 0.01, and 0.001).

Declines in maximum mitochondrial respiration were observed in 28‐month‐old WT mice compared to their 6‐month‐old counterparts, including ADP‐stimulated and uncoupled maximum respiration stimulated by FCCP, in isolated mitochondria from PL muscles (Figure 4A). Similar patterns were also noted in baseline (*p* = 0.070), oligomycin (*p* = 0.054), and antimycin (*p* = 0.062) states in WT mice, though not significant. Age‐related declines in mitochondrial respiration were noted in GHSR‐1a KO mice; these were only significant in baseline and ADP and FCCP‐stimulated maximum respiration (Figure 4A). Notably, KO mice showed a significantly reduced delta change (old vs. young) in the FCCP state compared to the WT mice (Figure S2A). Age‐related decreases were also noted in oxidative phosphorylation (OXPHOS) complex IV content in WT mice (Figure 4B,C). We quantified citrate synthase (CS) activity in isolated mitochondria and derived whole‐muscle CS activity by multiplying mitochondrial CS activity (μmol/ml/min/mg) by the total protein content (mg, evaluated by Bicinchoninic acid assay) of mitochondria isolated from PL muscles. Notably, older KO mice displayed greater CS activity than age‐matched WT mice, and a significant age‐related reduction in CS activity was observed only in WT animals (Figure 4D). We also measured cytochrome C protein levels in isolated mitochondria to assess mitochondrial health; 6 months old KO mice showed higher levels of cytochrome C than the same‐aged WT or 28 months old KO (Figure S2B). In PL muscles, the number of succinate dehydrogenase (SDH)‐positive cells, an indicator of oxidative capacity, decreased with age in WT mice. KO mice showed a similar age‐related decline, though it was not statistically significant (Figure 4E,F). In WT mice, the mitochondrial biogenesis marker peroxisome proliferator‐activated receptor gamma coactivator (PGC)‐1α was lower in 28‐month‐old WT mice compared to 6‐month‐old mice, with a tendency towards lower levels compared to the 24‐month group (*p* = 0.059). In contrast, 28‐month‐old KO mice exhibited higher PGC‐1α levels than their younger peers (*p* < 0.05 vs. 6 months; *p* = 0.060 vs. 24 months) and age‐matched WT mice (Figure 4G,I). Young KO mice showed a higher level of Sirtuin 1 (*Sirt1*), a potential regulator of PGC‐1α, than same‐aged WT (Figure 4G). To assess the regulator of mitochondrial content, we measured mitochondrial DNA (mtDNA) copy number in PL muscles from 6‐ and 28‐month‐old WT and KO mice (Quiros et al. 2017). We observed a significant age‐related decline in relative mtDNA expression in WT mice, whereas this reduction was not evident in the KO group (Figure 4G). We further evaluated several mitophagy markers, as they can be served for mitochondrial quantity control. In WT mice, the protein levels of the mitophagy marker p62 showed a mild decrease in muscle tissues at 24 and 28 months of age compared to the 6‐month group (*p* = 0.072 and *p* = 0.088, respectively). In contrast, p62 levels in KO mice increased at 24 months and returned to baseline levels by 28 months. Notably, p62 levels were lower in 6‐month‐old KO mice but higher in 24‐month‐old KO mice relative to their age‐matched WT counterparts (Figure 4H,I). Also, gene expression of *PINK1* (PTEN‐induced kinase 1), a key regulator of mitophagy, was significantly elevated in 28‐month‐old KO mice compared to WT mice of the same age (Figure 4H). In isolated mitochondria, 28‐month‐old KO mice displayed elevated protein levels of the mitophagy marker Parkin, normalized to voltage‐dependent anion channel 1 (VDAC1, Figure 4J), a marker of mitochondrial outer membrane. Consistently, 28‐month‐old KO mice also showed increased colocalization of autophagy marker microtubule‐associated proteins 1A/1B light chain 3B (LC3) and mitochondrial content marker Tomm20 compared to the same‐aged WT mice (Figure 4J).

### 2.5 Proteomic Analysis in Skeletal Muscle Reveals the Effects of GHSR‐1a Deletion on Correlations Between Kyoto Encyclopedia of Genes and Genomes (KEGG) Pathways and Sarcopenia Measures

To elucidate the molecular mechanisms underlying the progression of sarcopenia, we conducted proteomic analyses on GAS muscles of 6‐month‐old and 28‐month‐old WT and KO mice, in which we identified 3110 protein groups mapped from 24,483 detectable peptides. Individual protein abundances were quantified, and linear regression analyses were performed to assess associations between protein abundance and sarcopenia‐related measures, including grip strength, muscle mass, and treadmill running time. No significant correlations were detected at the individual protein level after multiple‐comparison adjustment (adjusted *p* < 0.05; data not shown).

**FIGURE 5:**
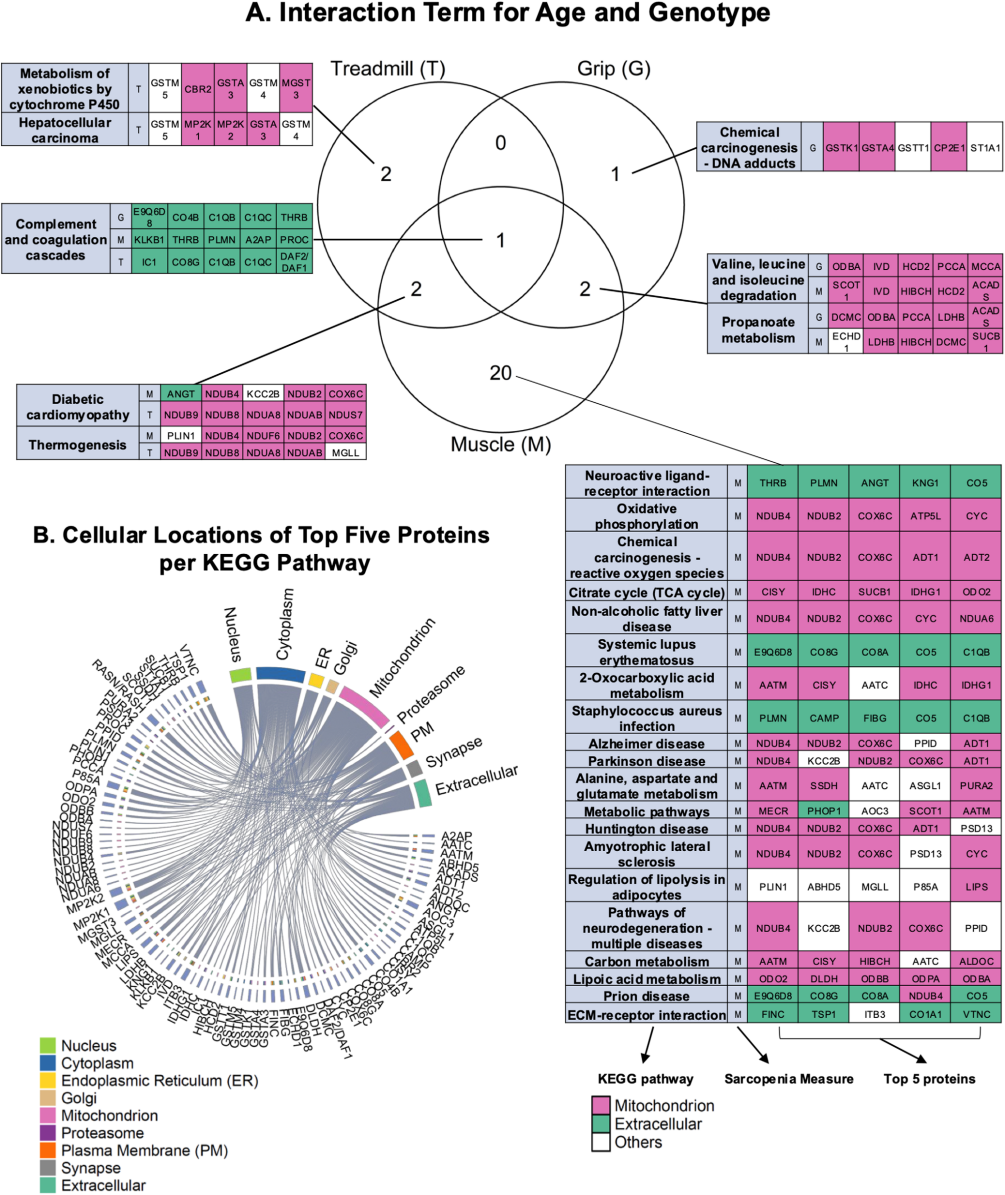
Proteomic analysis in skeletal muscles reveals the effects of GHSR‐1a deletion on correlations between Kyoto Encyclopedia of Genes and Genomes (KEGG) pathways and sarcopenia measures (treadmill running time [T], grip strength [G], and muscle mass [M]). (A) Venn diagrams show unique and shared KEGG pathways that correlate with treadmill running time, grip strength, or muscle mass, respectively, and that are perturbed by GHSR‐1a deletion with aging. Names of specific KEGG pathways are listed in the tables, together with the correlated sarcopenia measure (T, G, or M) and the top five proteins identified in each pathway. Proteins identified in the mitochondrion and extracellular region are highlighted in pink and green, respectively, while proteins from other cellular locations are shown in white. (B) The chord diagram demonstrates cellular locations of the top five proteins from each KEGG pathway, based on their Gene Ontology Cellular Component (GO‐CC) terms (see Table S2 for details).

To further identify pathways related to sarcopenia and evaluate how GHSR‐1a deletion affects these relationships differently in 6‐ and 28‐month‐old mice, we performed a competitive gene set test based on KEGG pathways (D. Wu and Smyth 2012). GHSR‐1a deletion had different effects on the correlations between KEGG pathways and sarcopenia measures in 6‐ and 28‐month‐old mice, with 5 pathways correlated with treadmill running time, 4 pathways correlated with grip strength, and 25 pathways correlated with muscle mass (Figure 5A and Table S1). Since KEGG pathway names do not always reflect the exact protein composition and several pathways share overlapping top‐ranked proteins, we listed the top five proteins from each pathway and mapped them to cellular locations using Gene Ontology (GO) Cellular Component terms (details in Table S2). As shown in Figure 5B, most of these proteins are located in mitochondria, followed by cytoplasm, plasma membrane (PM), and extracellular space (Figure 5B). Among the KEGG pathways, the complement and coagulation cascades pathway was affected by GHSR‐1a deletion differently in 6‐ and 28‐month‐old mice and was correlated with all three sarcopenia measurements, with its top‐ranked proteins located in the extracellular region (highlighted in green). Also, top‐ranked proteins correlated with treadmill performance and grip are primarily associated with the complement pathway and immune responses (IC1, CO8G, C1QB, C1QC, CO4B, and DAF), whereas those correlated with muscle mass are predominantly involved in coagulation and fibrinolysis (KLKB1, THRB, PLMN, A2AP, and PROC). Diabetic cardiomyopathy and thermogenesis pathways were found to be affected and correlated with treadmill and muscle mass, with most top‐ranked proteins being mitochondria‐based (highlighted in pink). Specifically, these proteins are primarily components of OXPHOS, including Complex I subunits (NDUB9, NDUB8, NDUA8, NDUAB, NDUS7, NDUB4, NDUB2, NDUF6) involved in ATP production, and Complex IV subunit COX6C, which facilitates the final step of mitochondrial respiration. Propanoate metabolism and valine, leucine, and isoleucine degradation pathways were affected in their correlations with grip strength and muscle mass. The top‐ranked proteins within these pathways are also mostly located in mitochondria, including those involved in amino acid metabolism (IVD, PCCA, MCCA, HIBCH), fatty acid beta‐oxidation (HCD2, DCMC, ACADS), the TCA cycle (ODBA, SUCB1, SCOT1), and glycolysis (LDHB).

As several of the top five proteins identified in the interaction term were localized to mitochondrion based on GO Cellular Component analysis, we further evaluated these proteins using GO Biological Process annotations. These mitochondrial proteins were predominantly related to mitochondrial respiration and ATP production, including aerobic respiration (GO:0009060), mitochondrial electron transport from NADH to ubiquinone (GO:0006120), proton motive force–driven mitochondrial ATP synthesis (GO:0042776), respiratory electron transport chain (GO:0022904), oxidative phosphorylation (GO:0006119), tricarboxylic acid cycle (GO:0006099) as well as mitochondrial ADP (GO:0140021) and ATP transmembrane transport (GO:1990544). While a subset of proteins was also associated with mitochondrial membrane function, oxidative stress responses, fatty acid and amino acid metabolism, and mitochondrial biogenesis, the majority were linked to mitochondrial respiration and energy metabolism (Table S3).

Additionally, as the second most populated top 5 protein location was extracellular, growing evidence shows its association with fibrosis and further leads to sarcopenia (Brack et al. 2007; Cai et al. 2023; Lieber and Ward 2013; Lofaro et al. 2021; Sanes 2003). We assessed muscle extracellular matrix (ECM) remodeling by quantifying collagen content in TA muscles using Masson's Trichrome staining, in which collagen is visualized in blue. The collagen‐positive area was quantified as a percentage of the total muscle cross‐sectional area. Aging significantly increased collagen content in both WT and KO mice; however, no significant genotype differences were observed (Figure S3A,B). To further explore the potential functional relevance of ECM alterations, we performed correlation analyses between collagen content and sarcopenia‐related outcomes, including muscle mass, grip strength, and treadmill running time, within the same age groups (Figure S3C). In young mice, collagen content was inversely correlated with grip strength (Spearman's correlation coefficient rho: −0.79, *p* = 0.036, *N* = 7). In contrast, in old mice, collagen content showed only a modest correlation with muscle mass though not significant (Spearman's correlation coefficient rho: −0.44, *p* = 0.057, *N* = 19), but no significant association with grip strength or treadmill performance.

### 2.6 GHSR‐1a Deletion Did Not Affect Survival

We conducted a Kaplan–Meier survival analysis on 267 WT and 222 KO male mice. The median survival age was 126 weeks for WT mice and 125 weeks for KO mice, with no significant difference in survival between the two genotypes (Figure S4).

### 2.7 PF‐5190457, a GHSR‐1a Inverse Agonist, Reduces Food Intake, Body Weight, and Body Fat While Increasing Endurance as Well as Mitophagy and Mitochondrial Biogenesis Markers in Skeletal Muscle in Male Mice

**FIGURE 6:**
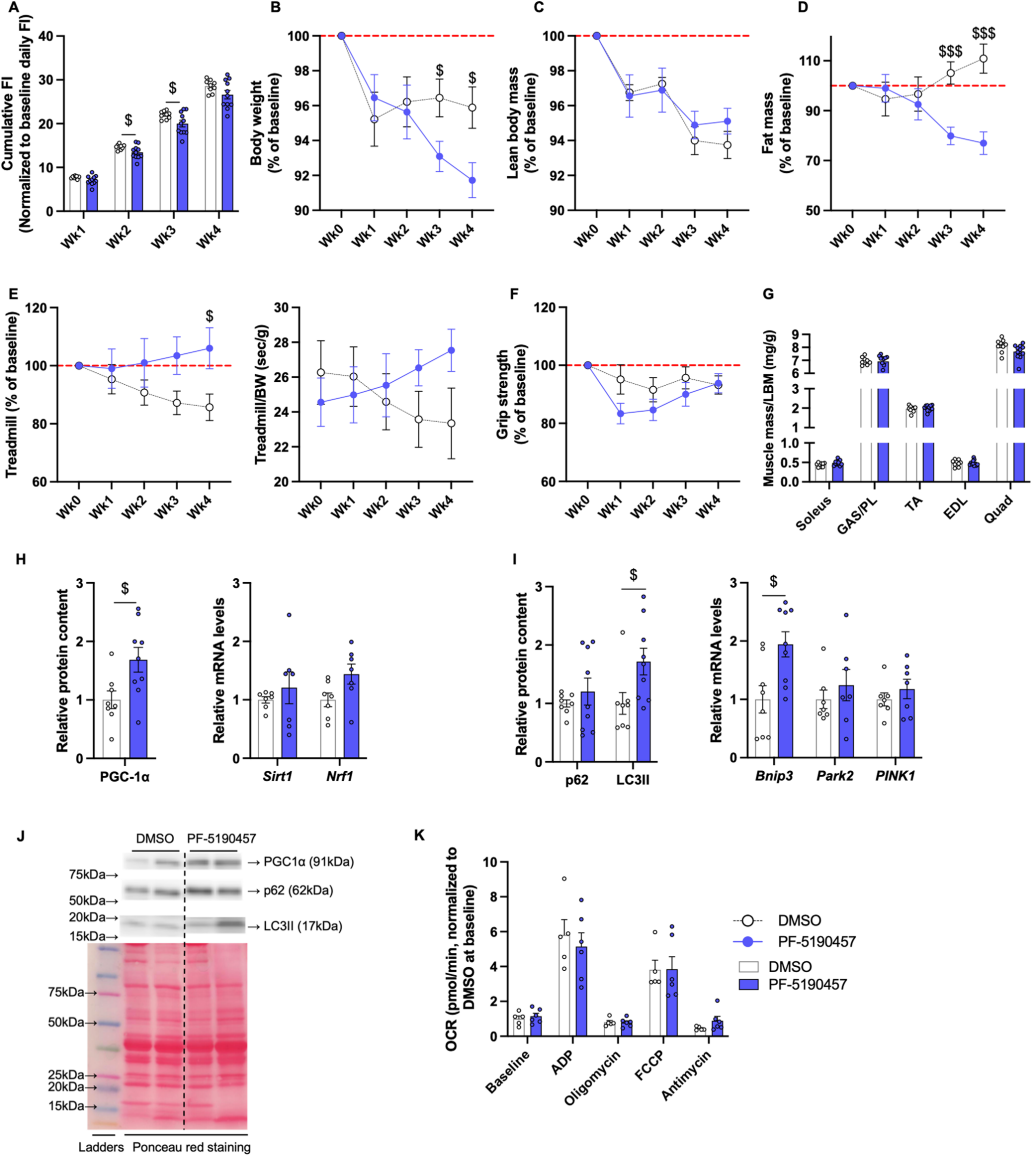
PF‐5190457, a GHSR‐1a inverse agonist, reduced food intake, body weight, and body fat, and improved endurance in 9–11 months old mice by increasing molecular markers of mitophagy and mitochondrial biogenesis in skeletal muscles. 9–11 months old C57BL/6J male mice were treated with either DMSO in saline or PF‐5190457 for 28 days. Weekly measurements include (A) cumulative food intake, normalized to baseline daily food intake (g/g), (B) body weight, (C) lean body mass (LBM), (D) fat mass, (E) treadmill running time, and (F) grip strength, all expressed as a percentage of baseline levels at week 0. (G) Individual muscle mass at the end of the treatment normalized to LBM (g/g). (H, I) Relative protein content of PGC‐1α, p62 and LC3II in GAS muscle, measured by Western blotting, quantified by densitometry, and normalized to Ponceau red signal, with (J) their representative Western blots and Ponceau red staining. mRNA expression of mitochondrial markers *Sirt1* and *Nrf1* (H), and mitophagy markers *Bnip3*, *Park2*, and *PINK1* (I), measured in GAS muscles by RT‐qPCR. *Hprt* was used as a reference gene, and data are expressed as the relative mRNA fold change from the DMSO‐treated group. (K) Mitochondrial respiration, measured as the oxygen consumption rate (OCR) at different states, including baseline, and after stimulation with ADP, oligomycin, FCCP, and antimycin, in isolated mitochondria from PL muscles (pmol/min). Relative OCR levels were normalized to the baseline state in the DMSO group. Independent *t*‐tests (two‐tailed) were performed to detect differences between treatments ($, $$, $$$: *p* < 0.05, 0.01, and 0.001). Data are presented as mean ± SE. Sample sizes: *N* = 9 (DMSO) and *N* = 11 (PF‐5190457) for panels A, B, D, E, F, and G; *N* = 7–9 for panels C, H, and I; *N* = 5 and 6 for panel K.

To investigate if pharmacologically targeting GHSR‐1a replicates the effects of GHSR‐1a deletion on muscle function and molecular markers, middle‐aged male mice (9–11 months) were treated with the GHSR‐1a inverse agonist PF‐5190457 (10 mg/kg, twice daily) in 10% DMSO for 28 days. Mice treated with 10% DMSO in saline served as the control group. Baseline measurements of food intake, body composition, and muscle function were taken before treatment and at weekly intervals until day 28. Weekly measurements were normalized to baseline levels and expressed as percentages. Decreases in cumulative food intake due to PF‐5190457 administration were observed at week 1 (*p* = 0.051) and reached significance in weeks 2 and 3 (Figure 6A). Body weight and fat mass were lower in PF‐5190457‐treated mice at weeks 3 and 4, while lean body mass remained unchanged throughout the treatment (Figure 6B–D). Improved treadmill running time in PF‐5190457‐treated mice started to show at week 3 (*p* = 0.059) and reached significance at week 4 (Figure 6E). After normalizing running time to body weight, the PF‐5190457 effect persisted, although it did not reach statistical significance (*p* = 0.081 at week 4, Figure 6E). Grip strength was not significantly different between groups (Figure 6F). At the end of the study, hindlimb skeletal muscles were collected for mass measurements. The normalized soleus muscles tended to be larger in PF‐5190457‐treated mice compared to DMSO‐treated mice (*p* = 0.087), whereas the normalized Quad tended to be larger in DMSO‐treated mice (*p* = 0.071). No significant differences were found in other muscles between groups (Figure 6G). Protein levels of the mitochondrial biogenesis marker PGC‐1α were elevated in the GAS muscles of PF‐5190457‐treated mice. Similarly, the transcript level of nuclear respiratory factor 1 (*Nrf1*) showed an increasing pattern with PF‐5190457 treatment (*p* = 0.056, Figure 6H). Markers of mitophagy, including LC3II protein and BCL2 Interacting Protein 3 (*Bnip3*) mRNA levels, were also increased following administration of PF‐5190457. Although no significant differences were detected in p62, *Park2*, and *PINK1*, their expression patterns were consistent with the observation in LC3II (Figure 6I). Mitochondrial respiration in isolated mitochondria in PL muscles from each group at the end of the study showed no significant difference across groups (Figure 6K).

Additionally, we examined the effects of PF‐5190457 on muscle mass and function in older mice (25–27 months of age). As in the younger cohort, these mice received either PF‐5190457 or DMSO for 4 weeks. Food intake, body composition, treadmill running time, and grip strength were assessed at baseline and weekly after the first dose (Figure S5A–F). Although no significant differences were detected across these parameters, PF‐5190457–treated mice showed lower cumulative food intake by week 3 (*p* = 0.081, Figure S5A) and exhibited numerically higher treadmill running times (Figure S5E). At the end of the study, hindlimb muscle mass was evaluated, and no significant difference was observed between treatments (Figure S5G). Notably, PF‐5190457 treatment resulted in significant increases in both basal and ADP‐stimulated maximal respiration in isolated mitochondria in PL muscles (Figure S5H).

## 3 Discussion

Our study highlights the potential of blocking GHSR‐1a as a therapeutic approach to combat sarcopenia. We demonstrated that GHSR‐1a deletion attenuated the severity of sarcopenia by improving endurance and grip strength without affecting muscle mass or longevity in 23–30 months old male mice; and these changes were associated with elevated markers of mitochondrial biogenesis and mitophagy. Additionally, 28‐day treatment with GHSR‐1a inverse agonist PF‐5190457 improved mitochondrial markers in both middle‐aged and old mice, and in middle‐aged mice, it increased endurance while reducing food intake and fat mass.

Previous studies demonstrate that ghrelin promotes muscle mass and strength in cancer cachexia, chronic kidney disease, and chronic heart failure (Barazzoni et al. 2017, 2010; Liu et al. 2021; Sirago et al. 2017; Tamaki et al. 2015). These conditions are characterized by severe muscle atrophy and adipose wasting driven by negative energy balance, which ghrelin mitigates by increasing food intake, restoring energy balance and exerting direct anabolic effects on skeletal muscles, including reducing inflammation, proteolysis, and autophagy while enhancing protein synthesis. Despite sharing symptoms with these muscle wasting conditions, sarcopenia is primarily driven by mitochondrial dysfunction rather than energy imbalance (Borsch et al. 2021; Campbell et al. 2022; Kerr et al. 2024). Interestingly, acylated ghrelin improves muscle strength but not endurance in aged mice, likely reflecting increases in lean mass rather than enhanced oxidative capacity (Guillory et al. 2017). Conversely, ghrelin deletion or treatment with unacylated ghrelin enhances oxidative metabolism and improves muscle mass and/or function in aging models, suggesting that ghrelin's anabolic actions in acute wasting conditions do not directly translate to sarcopenia (Agosti et al. 2020; Guillory et al. 2017; Kim et al. 2024). GHSR‐1a is not expressed in skeletal muscle, thus limiting investigation using a muscle‐specific KO model and indicating that ghrelin's effects on muscle are likely mediated indirectly through paracrine, central or systemic pathways (Sun et al. 2007). Despite the main orexigenic function of GHSR‐1a activation, we did not observe reduced food intake in GHSR‐1a KO mice, likely due to lifelong deletion and redundant pathways maintaining appetite. Thus, caloric restriction is unlikely to be an important factor in muscle function changes in KO mice; however, we later observed reduced food intake with a GHSR‐1a inverse agonist treatment. Although markers of nutritional status were not directly assessed in the present study, previous work demonstrated reduced circulating IGF‐1 levels in young (Liu et al. 2021), and lower glucose levels and improved insulin sensitivity in 20‐month‐old male mice with GHSR‐1a deletion (Lin et al. 2011; Sun et al. 2008).

Unlike GH‐deficient mouse models that exhibit extended lifespan (Aguiar‐Oliveira and Bartke 2018), GHSR‐1a deletion did not improve lifespan in the present study. This finding aligns with our previous work in ghrelin knockout mice, also with low systemic IGF‐1 levels but unaffected lifespan (Guillory et al. 2017). Notably, many studies have reported that lifespan regulation in mammals is driven primarily by alterations in GH signaling rather than by secondary or modest reductions in IGF‐1 alone (Bokov et al. 2011; Brown‐Borg 2022; Coschigano et al. 2003). These findings further support the hypothesis that a more significant suppression of the GH/IGF‐1 pathway, beyond the level achieved by inhibiting the ghrelin/GHSR‐1a axis, is required to elicit improvements in lifespan.

Here we report that aged KO mice preserved muscle strength and endurance compared to age‐matched WT mice despite having similar declines in muscle size. The enhanced muscle force production during fatigue tests but not peak force during force‐frequency tests suggests an improvement in muscle quality in aged KO mice through reduced muscle fatigability rather than increased muscle force. Skeletal muscle mitochondria play a central role in maintaining endurance and fatigue resistance in aging muscles. PGC‐1α is crucial in regulating mitochondrial biogenesis, supporting energy production during contraction and protecting muscle from oxidative stress, inflammation, and apoptosis (Z. Wu et al. 1999). Aging in both humans and rodents is associated with reduced PGC‐1α expression and mitochondrial content (Ghosh et al. 2011; Kang et al. 2013; Yeo et al. 2019). In C57BL/6J mice, PGC‐1α levels decline with sarcopenia and positively correlate with muscle mass, grip strength, and endurance (Kerr et al. 2024). Consistently, genetic modulation of PGC‐1α alters skeletal muscle oxidative capacity and endurance in aged mice (Gill et al. 2018; Handschin et al. 2007; Yang et al. 2020). In the current study, we demonstrate that aged GHSR‐1a KO mice exhibited higher levels of PGC‐1α in skeletal muscle compared to aged WT mice, suggesting that mitochondrial biogenesis may, at least in part, mediate their enhanced muscle function and decreased fatigability. PGC‐1α can be activated by SIRT1‐regulated deacetylation in skeletal muscles, which has been reported in exercise‐mediated activation of mitochondrial biogenesis. Consequently, PGC‐1α interacts with several nuclear transcription factors, including NRF‐1, NRF‐2 and estrogen‐related receptor alpha (ERRα), activating mitochondrial proteins such as mitochondrial transcription factor A (TFAM), which is responsible for mtDNA replication, transcription, and maintenance (Bhatti et al. 2017). Although *Sirt1* transcript levels mainly increased in young mice with GHSR‐1a knockout, this may partially explain the activation of Sirt1–PGC‐1α–mediated mitochondrial biogenesis and the blunting of age‐related mtDNA content decline in KO mice. Interestingly, we did not observe a significant effect of GHSR‐1a deletion on OXPHOS complexes in isolated mitochondria and only partially attenuated age‐related decline in uncoupled maximum respiration. These findings suggest that the effects of GHSR‐1a deletion on mitochondria are likely to occur at the whole‐muscle level by increasing mitochondrial content rather than improving mitochondrial function at the individual mitochondrion level, as supported by a milder decrease in CS activity with age in KO compared to WT. To further confirm changes in mitochondrial function at the whole‐muscle level, in situ mitochondrial respiration tests should be conducted in future studies. Deletion of GHSR‐1a improved mitophagy markers, including the protein level of p62 and the transcript level of *PINK1* in aged KO mice. Moreover, increased coexpression of mitophagy/autophagy and mitochondrial markers in aged KO mice was evidenced by the Parkin‐to‐VDAC ratio in isolated mitochondria and LC3–Tomm20 colocalization in myofibers. Collectively, these findings support the hypothesis that GHSR‐1a deletion preserves endurance and reduces fatigability during aging, and that possible mechanisms include improved mitochondrial biogenesis and mitophagy.

Our proteomic analysis showed how GHSR‐1a deletion impacted correlations between KEGG pathways and three sarcopenic measurements (muscle mass, treadmill running time, and grip strength) with aging. The complement and coagulation cascades were the common pathway perturbed across all three sarcopenia measurements, and the top‐ranked proteins in this pathway (IC1, CO8G, C1QB, C1QC, CO4B, and DAF) may contribute to muscle function by modulating immune response (Massart et al. 2020; Pena Palomino et al. 2023), muscle regeneration and repair (Naito et al. 2012; Tu and Li 2023; Wang, Smith, et al. 2023), and protection against complement‐mediated damage (Kaminski et al. 2006; Wenzel et al. 2005). The overlapping pathways between treadmill running time and muscle mass included diabetic cardiomyopathy and thermogenesis, with top‐ranked proteins closely linked to OXPHOS complexes, ATP production, and mitochondrial respiration. Grip strength and muscle mass, in contrast, were associated with propanoate metabolism and the degradation of valine, leucine, and isoleucine, with top‐ranked proteins involved in amino acid catabolism. However, these proteins remained primarily mitochondrial in localization and were involved in biological processes underlying oxidative phosphorylation, such as electron transport, proton translocation, ATP synthesis, and substrate metabolism. Together, these findings highlight the central role of mitochondrial components in maintaining muscle mass and function, with endurance predominantly supported by mitochondrial metabolism, whereas muscle strength is more dependent on amino acid–related pathways. Additionally, pathways perturbed by GHSR‐1a deletion during aging and correlated with muscle mass alone were mostly mitochondrial but also included many extracellular proteins, such as those in the extracellular matrix (ECM)–receptor interaction pathway. Growing evidence indicates that the ECM plays a crucial role in sarcopenia, not only by maintaining muscle structure but also by serving as a reservoir for metabolites that support cellular homeostasis (Ahmad et al. 2020; Lofaro et al. 2021). Age‐related changes in ECM result in increased fibrosis (Lieber and Ward 2013; Lofaro et al. 2021) and impaired differentiation and regeneration in skeletal muscles (Brack et al. 2007; Cai et al. 2023; Sanes 2003). In the current study, Masson's Trichrome staining revealed that collagen accumulation in skeletal muscle increased with aging and was inversely correlated with muscle mass in aged mice. These findings are consistent with our proteomic pathway analyses, which indicated that ECM‐related pathways were primarily associated with muscle mass rather than functional outcomes in aged mice. Accordingly, our current data suggest that ECM remodeling is unlikely to be a primary driver of functional improvement induced by GHSR‐1a deletion in aged mice. Nevertheless, age‐associated ECM remodeling is clearly evident, and future studies should consider targeting ECM alterations as a potential therapeutic strategy for sarcopenia.

PF‐5190457 attenuated ghrelin‐ or hexarelin‐induced food intake and was well‐tolerated in male rodents (Brehm et al. 2020; Leko et al. 2024). It has been shown to be safe and well‐tolerated in phase 1b clinical trials for alcohol use disorder (Burnette et al. 2022). We selected a dose of 10 mg/kg PF‐5190457, which has shown minimal effects on locomotor activities in rats and crosses the blood–brain barrier (Lee et al. 2020). We found that 28 days of PF‐5190457 treatment significantly reduced food intake and adiposity and increased treadmill endurance in middle‐aged mice (9–11 months old), with a similar pattern observed in older mice (25–27 months old). Although the PF‐5190457 effect on endurance persisted after normalizing running time to body weight, we cannot fully distinguish the direct contribution of reduced food intake to the observed changes in muscle function, as caloric restriction may improve age‐related muscle functional decline due to its effects on preserving muscle fiber integrity, reducing mitochondrial oxidative stress and DNA damage (Civitarese et al. 2007; Drew et al. 2003; Faitg et al. 2019; Kim et al. 2008). A pair‐feeding study will be necessary to address this in future work. Similar to GHSR‐1a deletion, PF‐5190457 increased mitochondrial biogenesis marker PGC‐1α and produced a mild improvement in its downstream transcription factor *Nrf1*. While GHSR‐1a deletion in aged mice altered p62 and *PINK1* levels, PF‐5190457 treatment primarily elevated LC3II and *Bnip3*. Similar to p62, Bnip3 is another selective autophagy receptor embedded in the outer mitochondrial membrane and can directly bind to LC3, facilitating autophagosome recruitment and mitochondrial clearance (Novak et al. 2010). These findings suggest that chronic GHSR‐1a deletion and acute pharmacological inhibition may engage different components of the mitophagy machinery depending on the context and duration of intervention. Because endurance primarily depends on mitochondrial function and oxidative capacity, whereas grip strength is also influenced by muscle mass and fast‐twitch fibers, the lack of synchronized changes between these two parameters is not unexpected, particularly given that PF‐5190457 did not alter muscle size (Plotkin et al. 2021). In older mice, although changes in endurance are less significant compared to middle‐aged mice, the effects of PF‐5190457 are more evident in mitochondrial respiration, especially in basal and ADP‐stimulated maximum respiration states. Due to limited compound availability and the invasive nature of twice‐daily dosing over extended periods, treatment was restricted to 1 month. Notably, the improvement in endurance was more pronounced in week 4 than at earlier time points in both age groups, suggesting that a longer treatment duration may yield greater beneficial effects of PF‐5190457 on muscle endurance beyond 28 days. Our results also suggest that early (preventive) administration of this compound may be more effective in preventing sarcopenia than its administration once the syndrome is already established. This will need to be confirmed in future studies. Nevertheless, these results further show that pharmacological blockade of GHSR‐1a produces effects comparable to genetic deletion, highlighting its potential to improve endurance as well as to increase markers of mitochondrial biogenesis and mitophagy.

This study has some limitations. The evidence supporting a role for ghrelin/GHSR‐1a in regulating mitochondrial function is mainly derived from studies in male mice (Agosti et al. 2020; Guillory et al. 2017; Liu et al. 2021; Ma et al. 2011), so we initiated our investigations in male mice. Future studies will include female mice, as sex‐specific mechanisms contribute to the development of sarcopenia (Kerr et al. 2024). Additionally, due to limited sample availability, some of our results include only one group of aged mice (28 months old). This restricts our ability to assess the effects of GHSR‐1a deletion across a broader age spectrum. With sarcopenia in aged mice and limited muscle tissue resources from individual muscles, we need to use different hindlimb muscles for various experiments. However, since most hindlimb muscles in mice are composed of type II fibers, except for the soleus, we consider the intermuscle molecular differences to be minor. We summarize the major findings for different muscles in Table S4. Due to limited resources, we were only able to assess mitochondrial respiration in isolated mitochondria using the Seahorse XFe24 system, which restricted the range of mitochondrial substrates evaluated. Future studies should leverage O2K technology, which would enable a more comprehensive assessment of substrate‐specific mitochondrial function (Pharaoh et al. 2023) and allow for a deeper investigation of sex‐specific differences (Ritterhoff et al. 2021). Moreover, mitophagy and mitochondrial biogenesis are dynamic processes, and the current study only assessed static markers. Future studies should include other methods to detect dynamic changes in these pathways including mitophagy flux. Despite these limitations, our study provides compelling evidence that the absence of GHSR‐1a can significantly improve muscle function in aged mice without affecting longevity. Furthermore, a similar effect on preserving endurance with the GHSR‐1a inverse agonist PF‐5190457 suggests that GHSR‐1a is a druggable target, although the exact mechanisms suggested by our data need to be confirmed.

## 4 Conclusion

In summary, our study demonstrates that deletion of GHSR‐1a alleviates age‐related declines in muscle function and increased severity of sarcopenia without affecting lifespan. Similar effects on endurance, as well as reduced adiposity, were observed in middle‐aged mice with pharmacological inhibition of GHSR‐1a. Improvements in endurance are associated with markers of mitochondrial biogenesis and mitophagy, though causality remains to be confirmed. This study provides a strong rationale for future research targeting GHSR‐1a negatively as a therapeutic strategy for sarcopenia.

## 5 Methods

### 5.1 Animals

The study used male growth hormone secretagogue receptor (GHSR)‐1a wild type (WT) and knockout (KO) mice on a C57BL/6J background, categorized as 6‐months (5–7 months), 24‐months (23–26 months), and 28‐months (27–30 months). The original Ghsr−/− mice, obtained from Dr. Roy Smith's laboratory, were backcrossed with the C57BL/6J strain for at least ten generations to minimize potential background genetic variations (Liu et al. 2021). The mice used in this study were descendants of these congenic lines and were bred in the Animal Research Facilities at the Veterans Affairs Puget Sound Health Care System. In this model, GHSR‐1a has been deleted from all tissues. It is worth noting that this receptor is not expressed in skeletal muscles (Sun et al. 2007). A total of 67 WT and 99 KO mice were evaluated in this study, and the experimental unit was each single animal. To minimize potential confounders, all mice were individually housed in the same room, maintained on a 12/12 light/dark cycle (lights on at 6 AM), and acclimated to their environment and human handling for 1 week before experiments (day 0–6). Daily food intake was then evaluated for 5 days after acclimation (day 7–12). In the following week, mice underwent a treadmill test (day 14), grip test (day 16), and body composition (day 18) analysis via nuclear magnetic resonance (NMR) as described previously (Kerr et al. 2024; Liu et al. 2021). We used CO_2_ euthanasia for direct tissue harvest, whereas mice undergoing procedures requiring immediate functional assessment (e.g., Aurora muscle physiology or mitochondrial isolation from fresh tissue) were anesthetized with isoflurane followed by cervical dislocation. Hindlimb muscles were collected for mass estimation and biochemical analysis. Muscles for Western blotting, proteomics or mtDNA analysis were flash‐frozen in liquid nitrogen and stored at −80°C. Tissues for RNA extraction were preserved in RNAlater and stored at −80°C. Muscles designated for histology were embedded in OCT and flash‐frozen in liquid nitrogen–prechilled isopentane before storage at −80°C. Samples for mitochondrial isolation were processed immediately after collection. The specific muscles used for each assay are summarized in Table S4. Survival analysis was conducted on WT and KO mice that were born within the same 2‐year period. All procedures were approved by the Institutional Animal Care and Use Committee (IACUC) at the VA Puget Sound Health Care System and adhered to NIH Guidelines for the Use and Care of Laboratory Animals (IACUC protocol #0950).

### 5.2 Treadmill Test

Treadmill tests were conducted using the Exer‐6 M treadmill (Columbus Instruments, Columbus, OH). Mice were allowed to acclimate for 5 min on the stationary treadmill before testing. The protocol began at a speed of 5 meters (m) per minute for 5 min, with the speed increased by 1 m per minute thereafter. Mice were encouraged to run by gently tapping their rear with a cotton swab until exhaustion. A mouse was considered exhausted, and the test was concluded if, after three attempts to encourage it to run by tapping, it still refused and remained near the tester at the end of the treadmill. The protocol and volitional fatigue criterion were adapted from Kwak et al. (2020). The total time (seconds) the mouse remained on the treadmill was recorded.

### 5.3 Grip Strength

Grip strength is the most widely accepted method to estimate muscle strength, allowing for more generalizable results. It has also been used as one of the criteria for sarcopenia diagnosis by our group (Kerr et al. 2024) and others (Borsch et al. 2021; Xie et al. 2021). A grip strength meter with a digital force gauge was used to measure forelimb grip strength (Columbus Instruments, Columbus, OH). Mice were acclimated to the environment before grip strength testing by placing them on the gripping grid without applying any pulling force. The mouse was allowed to use only its forelimbs to grasp a pull bar connected to a force gauge to assess forelimb grip strength. The maximum grip strength was recorded on the force gauge in kilograms. The test was performed three times at 1‐min intervals. Maximum grip strength was recorded. Absolute grip strength (g), and grip strength normalized to body weight (g/g) or hindlimb muscle mass (g/g) were reported.

### 5.4 Evaluation of Sarcopenia Status

Sarcopenia status was evaluated using three clinically relevant criteria adapted for mice: muscle strength, muscle mass, and physical performance. Muscle strength was assessed by forelimb grip strength (g), muscle mass by total hindlimb muscle weight (mg), and physical performance by treadmill running time (seconds to exhaustion). Cutoff values for each parameter were defined as 2 standard deviations below the mean of a young adult male C57BL/6J reference cohort, adapted from our previous study (Kerr et al. 2024). Specifically, low grip strength was defined as < 131 g, low muscle mass as < 708 mg, and impaired physical performance as < 514 s. Mice were then classified as nonsarcopenic (non‐S, no deficits), probable sarcopenic (PS, 1 deficit), sarcopenic (S, 2 deficits), or severe sarcopenic (SS, 3 deficits), depending on the number of criteria met.

### 5.5 GHSR‐1a Inverse Agonist Experiment

Male C57BL/6J mice aged 9–11 months (middle‐aged) and aged 25–27 months (old, age at week 0) were included to test the effects of the GHSR‐1a inverse agonist PF‐5190457. Baseline measurements were conducted at week 0 after the mice had been individually housed for 5 days in the same room for acclimation. These measurements included food intake, body weight, body composition, treadmill running time, and grip strength. Animals were nonsystematically assigned to one of the following groups: the treatment group (PF‐5190457) received 10 mg/kg PF‐5190457 subcutaneously twice daily for 28 days (ghrelin receptor inverse agonist, MedChemExpress) in 10% DMSO in saline, while the control group received 10% DMSO in saline at a volume adjusted to match the body weight‐to‐liquid ratio used in the PF‐5190457 group. Throughout the experiment, food intake and body weight were measured daily, while body composition, treadmill tests, and grip tests were conducted weekly. Group allocation and data analysis were performed by one researcher, while treatment administration and behavioral testing were conducted by two researchers in a blinded manner using coded identifiers. At the conclusion of the 28‐day dosing, the mice were euthanized for tissue collection as previously described. Gastrocnemius muscles from middle‐aged mice were used to identify molecular markers. Plantaris muscles from both middle‐aged and old mice were used to determine mitochondrial respiration. No animals were excluded at the end of the experiment.

### 5.6 In Situ Muscle Physiology

An in situ muscle physiology test was performed on the TA muscle using the Aurora 1300A 3‐in‐1 Whole Animal System for Mice (Aurora Scientific, Ontario, Canada), as previously described (Kerr et al. 2024). A pair of needle electrodes was used to stimulate the TA muscle, with the electrodes placed directly on the deep side of the muscle's proximal end while the mouse was under isoflurane‐induced anesthesia. Optimal length (Lo, mm) was determined through single twitch tests, followed by tetanus (150 Hz) and force‐frequency tests (10, 30, 50, 75, 100, 125, 150, 200 Hz) at one‐minute intervals. Peak force (Po) was measured as the highest force production during the force‐frequency test. The physiological cross‐sectional area (pCSA) was calculated using the equation: pCSA (mm^2^) = muscle mass (mg) / [1.06 × Lo (mm) × 0.6], where 1.06 represents skeletal muscle density and 0.6 is the TA length‐to‐fiber ratio (Brooks and Faulkner 1988; Burkholder et al. 1994). Specific force (SPo) was then calculated by dividing peak force by the pCSA. Fatigue tests were conducted by stimulating the muscle at 150 Hz every 2 s for 4 min.

### 5.7 Immunohistochemistry, Succinate Dehydrogenase (SDH), and Masson's Trichrome Staining

The cross‐sectional area (CSA) of individual fibers from plantaris (PL) muscle was determined as previously described (Kerr et al. 2024, 2023). Plantaris was selected for fiber typing due to its mixed fiber composition and established use in previous studies (Bloemberg and Quadrilatero 2012). The muscle was mounted in OCT and sectioned into 10 μm slices at −22°C using a Cryostat (Leica CM3050S, Nußloch, Germany). After being dehydrated for 30 min, the muscle sections were blocked with 10% normal goat serum (NGS) in Phosphate Buffered Saline (PBS) for 1 h. The sections were then incubated with the following primary antibodies at room temperature for 1 h: SC‐71 (1:300) and BF‐F3 (1:25) to detect myosin heavy chain (MHC) isoforms IIA and IIB respectively (Developmental Studies Hybridoma Bank, Iowa City, IA), along with anti‐dystrophin to detect membranes (1:100, ab15277, Abcam). After three washes in PBS, sections were incubated for 1 h with the corresponding secondary antibodies (Thermo Fisher Scientific, Waltham, MA): Goat anti‐Mouse IgG1, Alexa Fluor 488 (A21121, 1:500) for MHC‐IIA; Goat anti‐Mouse IgM (Heavy chain), Alexa Fluor 555 (A21426, 1:500) for MHC‐IIB; and Goat anti‐Rabbit IgG (H + L), Alexa Fluor Plus 647 (A32733, 1:200) for membranes. After another three washes in PBS, the sections were mounted with Prolong Gold AntiFade reagent (P36934, Thermo Fisher Scientific). A Nikon Ni‐E microscope equipped with NIS‐Elements software (Nikon, Tokyo, Japan) was used to capture 10× images of the PL muscle cross‐sections. Approximately 100 type IIA, 200 type IIB, and 60–80 type nonstained fibers from the entire PL area were analyzed to determine the muscle fiber CSA, as previously described (Kerr et al. 2024). The percentage of each fiber type in the PL muscles was determined using the Cell Counter plugin in ImageJ analysis software (National Institutes of Health, http://rsb.info.nih.gov/ij/). To identify colocalization of Tomm20 and LC3 in tibialis anterior (TA) muscles, primary antibodies (Anti‐TOMM20 antibody, ab56783, Abcam, and LC3B (E7X4S) XP Rabbit mAb, 43566 T, Cell Signaling) were used, followed by corresponding secondary antibodies. The entire TA area was imaged at 40× using the Nikon Ni‐E's stitching function. A colocalization plugin in ImageJ was used to quantify Tomm20/LC3‐positive cells.

The SDH staining protocol was performed as previously described (Kerr et al. 2024). Plantaris (PL) muscle sections were incubated for 30 min at 37°C in a solution containing 10 mL of 0.2 M phosphate buffer (pH 7.6), 270 mg of sodium succinate (S2378, Sigma), and 10 mg of nitro blue tetrazolium (NBT, N6876, Sigma). After incubation, sections were washed three times with dH2O to remove unbound NBT, followed by three washes in acetone solutions of increasing concentration (30%, 60%, and 90%) to ensure thorough removal. The sections were then rinsed with dH2O and mounted with an aqueous mounting medium. Three trained and blinded researchers quantified the whole PL section using ImageJ (Fiji) software.

Masson's Trichrome stain was performed to assess collagen levels in TA muscles. A Trichrome Stain (Connective Tissue Stain) Kit (ab150686) from Abcam was used, and the procedure followed the manufacturer's instructions. Whole TA sections were imaged at 10× using the Nikon Ni‐E's stitching function. The “Color Deconvolution2” plugin was installed in Image J, and the “Masson Trichrome” vector was selected to quantify collagen‐positive area in blue. The percentage of collagen‐positive area in the whole TA muscle was calculated as the ratio of collagen‐positive area to total TA area in ImageJ (Fiji).

### 5.8 Real‐Time Reverse Transcription‐Quantitative Polymerase Chain Reaction (RT‐PCR)

Quad (aging cohorts) or GAS muscles (PF‐5190457 interventions) were preserved in RNAlater (Qiagen) after collection. RNA was isolated using the Qiagen RNeasy mini kit, and transcription levels were quantified with the BioTek Cytation 5. cDNA synthesis was performed using the QuantiTect Reverse Transcription Kit (Qiagen). RT‐PCR was conducted on an ABI 7500 instrument using TaqMan Expression Assays (Thermo Fisher). *Bnip3, Sirt1, PINK1, Nrf1, and Park2* gene expression were normalized to *Hprt* and expressed as relative fold‐change using the 2^−ΔCT^ method.

### 5.9 Mitochondrial DNA (mtDNA)

Approximately 15 mg of the PL muscle was used to isolate DNA with the QIAamp DNA Mini Kit (Qiagen). Transcription levels were measured using the BioTek Cytation 5. Quantitative real‐time PCR was performed with 100 ng of DNA, predesigned primers, SYBR Green master mix, and an ABI 7500 instrument to detect mtDNA depletions. mtDNA gene 16S was normalized to the nuclear gene Hexokinase 2 (HK2) and expressed as the relative mtDNA to nDNA copy number ratio using the 2‐ΔCT method. The following primers were purchased from Thermo Fisher Scientific: 16S rRNA: Forward: 5′‐CCGCAAGGGAAAGATGAAAGAC‐3′; Reverse: 5′‐TCGTTTGGTTTCGGGGTTTC‐3′; HK2: Forward: 5′‐GCCAGCCTCTCCTGATTTTAGTGT‐3′; Reverse: 5′‐GGGAACACAAAAGACCTCTTCTGG‐3′.

### 5.10 Western Blotting

Protein extraction from whole muscle, protein quantification, and electrophoresis were described previously (Kerr et al. 2024). OXPHOS complex, Parkin, VDAC1, and cytochrome C protein levels were evaluated in isolated mitochondria as described in the following session (Mitochondria isolation). In brief, the BIO‐RAD Criterion Cell system was used to separate 50 μg of whole muscle homogenate or 10 μg of isolated mitochondria per lane on NuPAGE Bis‐Tris Midi Protein Gels, 4%–12% (WG1402A, Thermo Fisher Scientific). Kaleidoscope or Precision Plus Protein Standards (1610375 and 1610374, Bio‐Rad) were loaded at both ends of the membrane for reference. Proteins were transferred to nitrocellulose or PVDF membranes using a BIO‐RAD Criterion Blotter (170–4070, BIO‐RAD) or XCell4 SureLock Midi‐Cell (WR0100, Thermo Fisher Scientific). Nitrocellular membranes were stained with Ponceau S (59803, Cell Signaling), imaged, and cut based on molecular weight. After blocking in 5% nonfat dry milk, membranes were incubated overnight at 4°C with the following primary antibodies: anti‐SQSTM1/p62 (1:1000, Ab56416, Abcam), anti‐PGC1 alpha (1:1000, ab191838, Abcam), LC3B (1:500, NB100‐2220 Novus), total OXPHOS Rodent WB Antibody Cocktail (1:1000, ab110413, Abcam), anti‐Parkin antibody (1:500, ab77924, Abcam), anti‐VDAC1 (1:1000, ab14734, Abcam), and Cytochrome c Antibody (1:1000, 4272, Cell Signaling). After washing, membranes were incubated with HRP‐conjugated secondary antibodies for 1 h at room temperature. Detection was performed using SuperSignal West Dura Substrate (34075, Thermo Fisher Scientific) and imaged with ImageQuant LAS 4000 (GE Health Care). Densitometry was analyzed using ImageJ and normalized to the Ponceau red signal.

### 5.11 Mitochondria Isolation and Mitochondrial Respiration Measurements

Mitochondria were isolated from freshly harvested PL muscles using a modified protocol (Boutagy et al. 2015; Rogers et al. 2011) with mitochondria isolation buffer (MIB: 210 mM sucrose, 2 mM EGTA, 40 mM NaCl, 30 mM HEPES, pH 7.4) on ice. Protein concentration was quantified using the BCA method, and the mitochondria pellet was resuspended in mitochondrial assay solution (MAS: 70 mM sucrose, 220 mM d‐mannitol, 10 mM KH2PO4, 5 mM MgCl2, 2 mM HEPES, 1 mM EGTA, 0.2% fatty‐acid free BSA, pH 7.4) with substrates (5 mM malate, 5 mM pyruvate). The sample (7.5 μg/well) was loaded in triplicate, and the oxygen consumption rate (OCR) was measured in real time using an XFe24 Seahorse Analyzer (Agilent Technologies). Sequential injections included ADP (2 mM), oligomycin (2 μM), FCCP (4 μM), and antimycin A (2 μM).

### 5.12 Citrate Synthase Activity

Citrate synthase (CS) activity in isolated mitochondria from plantaris (PL) muscle was measured using the Citrate Synthase Activity Kit (CS0720, Sigma) according to the manufacturer's instructions. For each assay, 3 μg of isolated mitochondrial protein was analyzed in duplicate. CS activity was normalized to protein load and expressed as μmol/mL/min/mg. To estimate total CS activity in the whole PL muscle, the normalized CS activity (μmol/mL/min/mg) was multiplied by the total mitochondrial protein content determined by BCA assay and expressed as μmol/mL/min.

### 5.13 Proteomic Analysis

Gastrocnemius muscles from male WT and KO mice aged 6–7 months and 27–29 months were used for proteomic analysis (*N* = 6/group). Batch design was randomly balanced based on condition ratios. The samples were divided into 2 batches of 12 individual samples and 2 pooled references for a total of 14 samples per batch. A pool of all 24 samples was used to create a reference pool to be used as a common reference, which was homogenized, aliquoted, frozen, and used to compare between the two batches. The processes of sample lysis and digestion, liquid chromatography and data‐independent acquisition (DIA) mass spectrometry, DIA signal processing were described previously (Kerr et al. 2024). Briefly, peptide identification and quantification were performed using chromatogram libraries generated from pooled reference samples, and peptide fragment ion intensities were integrated and summed to derive relative protein abundance across samples. Linear regression analyses were then carried out for all mice to explore potential correlations between individual proteins and any of the sarcopenia measures, including grip strength, muscle mass, or treadmill running time (*p* < 0.05). To further identify pathways that may be related to sarcopenia and evaluate how GHSR‐1a deletion affects these relationships differently in 6‐ and 28‐month‐old mice, we performed a competitive gene set test based on KEGG pathways using the ‘camera’ function from the Bioconductor limma package in R. This method can perform competitive gene set testing using any linear model and adjusts for interprotein correlations to reduce bias (Wu and Smyth 2012). We fit a model that included an interaction term between individual sarcopenia measurement (treadmill, muscle mass or grip strength), age group, and genotype and performed the gene set test based on the *t*‐statistic for the interaction term (False discovery rate, FDR < 0.05). For the top five proteins from each KEGG pathway, gene names were submitted to the UniProt ID mapping tool (https://www.uniprot.org) to retrieve Gene Ontology Cellular Component (GO‐CC) annotations and assign cellular locations. Mitochondrial proteins were further analyzed by GO Biological Process. The chord diagram demonstrating the cellular localization of proteins was generated using the R packages circlize and ComplexHeatmap (Gu 2022; Gu et al. 2014). The Skyline documents, raw files for quality control and DIA data are available at Panorama Public. ProteomeXchange ID: PXD048723. Access URL: https://panoramaweb.org/mouse‐gastroc‐sarcopenia‐proteomics.url.

### 5.14 Statistics

The sample size was determined based on our previous study investigating the effects of aging on WT mice, where the sample size provided sufficient statistical power to detect significant differences in the same outcome measurements as those described in the current study (Kerr et al. 2024). Two‐way analysis of variance (ANOVA) was performed to identify differences between genotypes (WT vs. KO) across age groups (6, 28 and/or 24 months) followed by Tukey post hoc test if there were three age groups (*p* ≤ 0.05) or LSD post hoc test if there were two age groups (*p* ≤ 0.05). For data not normally distributed based on the Shapiro–Wilk normality test (*p* < 0.05), nonparametric tests (Mann–Whitney) were performed for pairwise comparisons. Pearson Chi‐square tests were used to detect genotype differences across the rates of sarcopenic status in aged mice. An independent *t*‐test (two‐tailed) was used to compare the treatment effects of PF‐5190457 (vs. DMSO). Log‐rank (Mantel‐Cox) test was used to detect genotype difference in survival (*p* < 0.05). *p*‐values were reported and discussed if greater than 0.05 and less than 0.1 for abovementioned tests. Values are presented in mean ± SE. No data points were excluded from analysis. All statistical testing was performed using IBM SPSS version 18 software and GraphPad Prism 10. Statistics for proteomics are described in the Proteomic analysis. Data is available upon request to the corresponding author and first author.

## Author Contributions

H.L.K. and J.M.G. conducted the study design. H.L.K., K.K., N.M., L.C., G.E.M., J.W.M., T.K.B., D.J.M., and J.M.G. performed data analysis, interpretation, and manuscript preparation. H.L.K., K.K., N.M., B.A., and G.E.M. performed biochemical experiments and data collection. H.L.K., K.K., N.M., and E.D. conducted animal behavioral tests. A.R., E.D., B.I., S.J., L.C., T.L., A.C., R.B., and M.S. assisted in terminal surgeries and performed immunohistochemical quantification. M.J.M. provided guidance and resources for proteomic analysis. All authors reviewed and approved the final version of the manuscript.

## Funding

This work was supported by the U.S. Department of Veterans Affairs (BX002807, to J.M.G.), the Congressionally Directed Medical Research Programs (PC170059, to J.M.G.), and the NIH (R01CA239208, R01AG061558, both to J.M.G.). J.M.G. also received a Pilot Grant from the UW Nathan Shock Center supported by grant P30AG013280 from the National Institute on Aging. H.L.K. is funded by a New Investigator Award from the UW Diabetes Research Center and a Career Development Award from NIAMS (K01AR080787). L.C. is supported by the University of Washington T32 Training Grant, Division of Metabolism, Endocrinology and Nutrition (DK007247 to L.C.). E.D., M.S., and J.L. were supported by the VA Office of Research & Development Summer Research Program (SRP‐020‐22S). Core services provided by UW Diabetes Research Center and UW Nutrition Obesity Research Center are supported by grants (P30 DK017047) and (P30 DK035816) from the National Institute of Diabetes and Digestive and Kidney Diseases.

## Ethics Statement

All experiments were conducted with the approval of the Institutional Animal Care and Use Committee at VA Puget Sound Health Care System (Protocol no. 0950) and in compliance with the NIH Guidelines for Use and Care of Laboratory Animals.

## Conflicts of Interest

U.S. Patent Application #63192490 Compositions and Methods of Use of GSHR (J.M.G.).

## Supporting information


**Data S1:** WB—GHSR aging paper FINAL.

**Figure S1:** GHSR‐1a deletion did not affect water content. Body free water content was evaluated using NMR in 6‐ and 28‐month‐old male GHSR‐1a wild‐type (WT) and knockout (KO) mice. ***difference between age groups with the same genotype (*p* < 0.001). Data are shown as mean ± SE. Sample sizes: *N* = 21, 11, 21, 40 (left to right bars).
**Figure S2:** (A) Percent change (Δ, old vs. young) in each mitochondrial respiration state. $ indicates a significant difference between genotypes (*p* < 0.05). *N* = 11 (WT) and 20 (KO). (B) Cytochrome C levels in isolated mitochondria from 6‐ and 28‐month‐old male GHSR‐1a WT and KO mice. $ indicates a genotype difference within the same age group (*p* < 0.05). * indicates an age difference within the same genotype (*p* < 0.05). Data are shown as mean ± SE. Sample size: *N* = 6–8.
**Figure S3:** Collagen content in TA muscles from 6‐ and 28‐month‐old male GHSR‐1a wild‐type (WT) and knockout (KO) mice evaluated by Masson's trichrome staining. (A) Quantification of collagen‐positive area is expressed as a percentage of the whole muscle cross‐sectional area. *age difference between the same genotype (*p* < 0.05). Data are shown as mean ± SE. Sample sizes: *N* = 4 for 6 m groups and *N* = 11–12 for 28 m groups. (B) Representative images of Masson's trichrome staining (collagen area was stained in blue). (C) Spearman's correlation was used to assess the correlation between collagen‐positive area and each sarcopenic measurement (grip strength, treadmill running time, or muscle mass).
**Figure S4:** GHSR‐1a deletion did not affect survival. Weeks of survival in GHSR‐1a WT and KO male mice were assessed by Kaplan–Meier survival analysis (sample sizes are indicated in parentheses within legend). Differences between genotypes were analyzed using the Log‐rank (Mantel‐Cox) test (*p* < 0.05). *N* = 267 and 222 for WT and KO, respectively.
**Figure S5:** Effects of PF‐5190457 on food intake, body composition, muscle mass and function, and mitochondrial respiration in aged mice. 25–27‐month‐old C57BL/6J male mice were treated with either DMSO in saline or PF‐5190457 for 28 days. Weekly measurements include (A) cumulative food intake, normalized to baseline daily food intake (g/g), (B) body weight, (C) lean body mass (LBM), (D) fat mass, (E) treadmill running time, and (F) grip strength, all expressed as a percentage of baseline levels at week 0. (G) Individual muscle mass at the end of the treatment was normalized to LBM (g/g). (H) Mitochondrial respiration, measured as the oxygen consumption rate (OCR) at different states, including baseline, and after stimulation with ADP, oligomycin, FCCP, and antimycin, in isolated mitochondria from PL muscles (pmol/min). Relative OCR levels were normalized to the baseline state in the DMSO group. Independent *t*‐tests (two‐tailed) were performed to detect differences between treatments ($, $$: *p* < 0.05 and 0.01). Trend differences were detected at 0.05 ≤ *p* < 0.1 and are shown in the graph. Data are presented as mean ± SE. Sample sizes: *N* = 3 (DMSO) and *N* = 5 (PF‐5190457) for panel A, *N* = 6 for other panels.
**Table S1:** Kyoto Encyclopedia of Genes and Genomes (KEGG) Pathways Correlated with Each Sarcopenia Measure Significantly Perturbed by GHSR‐1a Deletion and Aging.
**Table S2:** Gene Ontology Cellular Component Categories for Top 5 Proteins Detected in Interaction Term.
**Table S3:** Gene Ontology Biological Process for Top 5 Mitochondrial Proteins Detected in Interaction Term.
**Table S4:** Muscle used for each figure.
**Table S5:** Absolute values and effect sizes (with 95% CI) of weekly measures in middle‐aged male mice with DMSO or PF‐5190457 treatment.
**Table S6:** *p*‐values of statistical analysis.

## Data Availability

Values for all data points associated with the manuscript and supplemental results are provided in the Supporting Data Values document. Data availability for proteomics is provided under “Proteomic analysis”. Additional information and detailed methods in this paper will be available upon direct request from the authors.
