# Supplementary material for: Ghrelin Receptor Deletion or Pharmacological Inhibition Improves Muscle Function in Aging Male Mice
Source: Aging Cell. 2026 Apr 15;25(4):e70472. doi: 10.1111/acel.70472 (PMC13083231; doi:10.1111/acel.70472)

Western blots for

“Ghrelin Receptor Deletion or Pharmacological Inhibition Improves Muscle Function in Aging Male Mice”

Haiming L. Kerr[1], Kora Krumm[1], Nornubari Myree[1], Artur Rybachok[1], Elizabeth Dacek[1], Brynn Irwin[1], Siyi Jiang[1], Lucas Caeiro[1], Barbara Anderson[1], Theresa Li[1], Amanda Chen[1], Ross Burnside[1], Jessica Li[1], Morgan Sydor[1], David J. Marcinek[2], Gennifer E. Merrihew[3], James W. MacDonald[4], Theo K. Bammler[4], Michael J. MacCoss[3], Jose M. Garcia*[1]

Full unedited blots (Figure 4B – data; Figure 4C – representative image)
OXPHOS complexes in isolated mitochondria

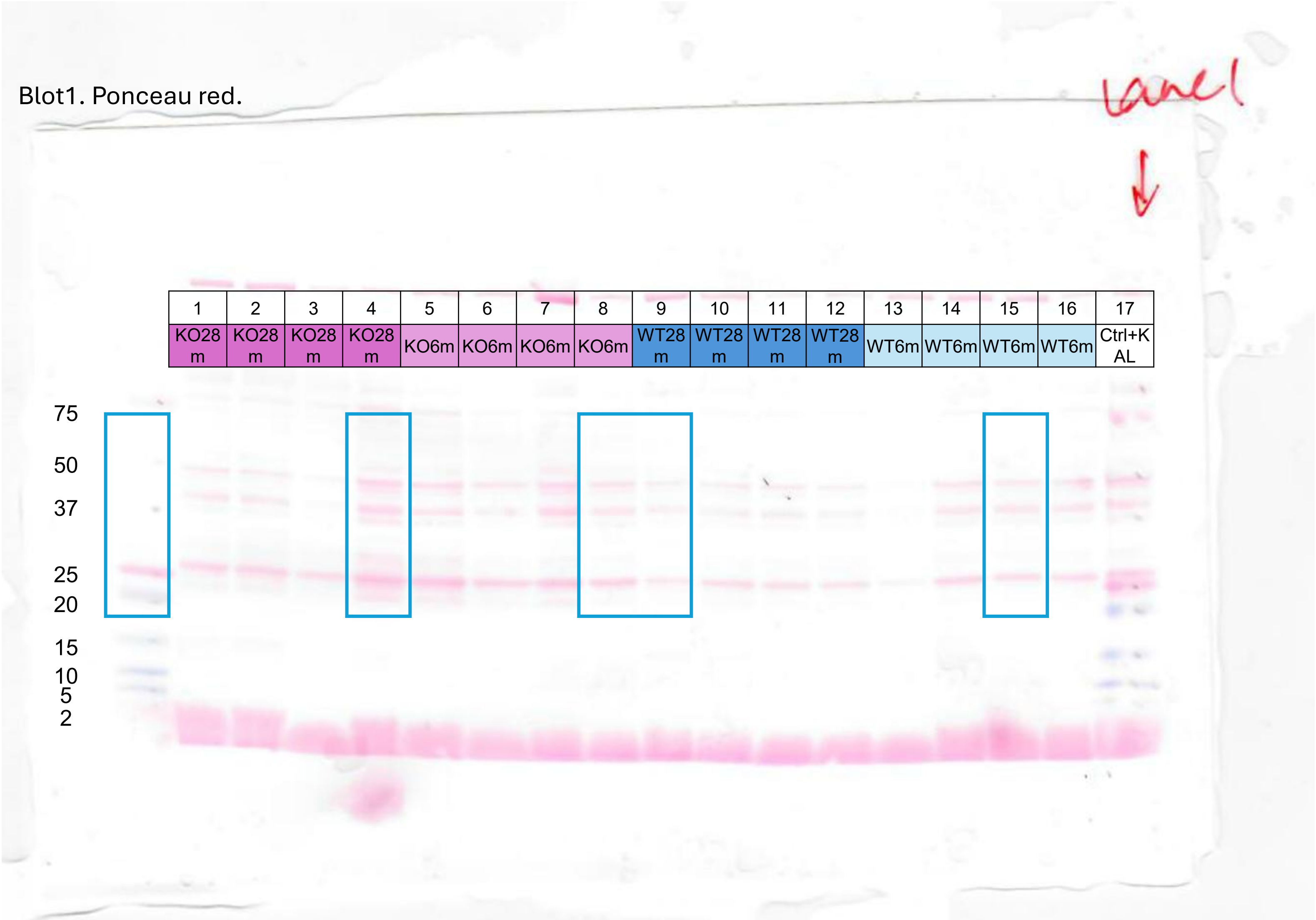

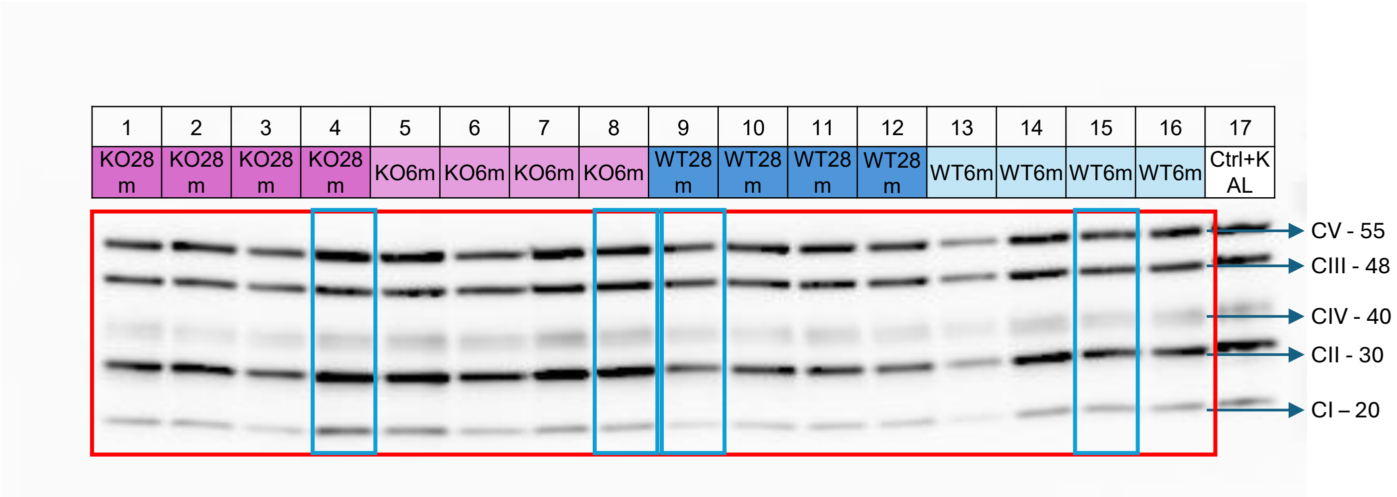

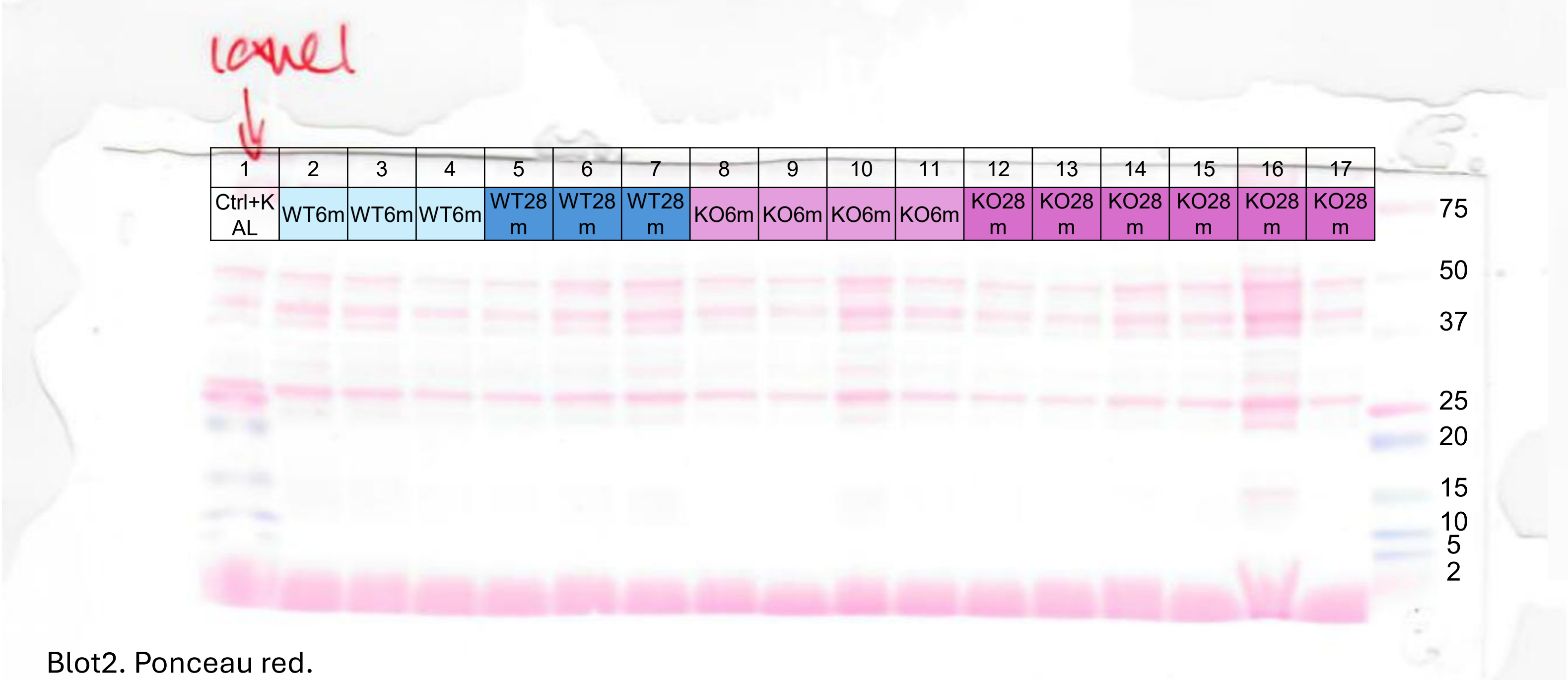

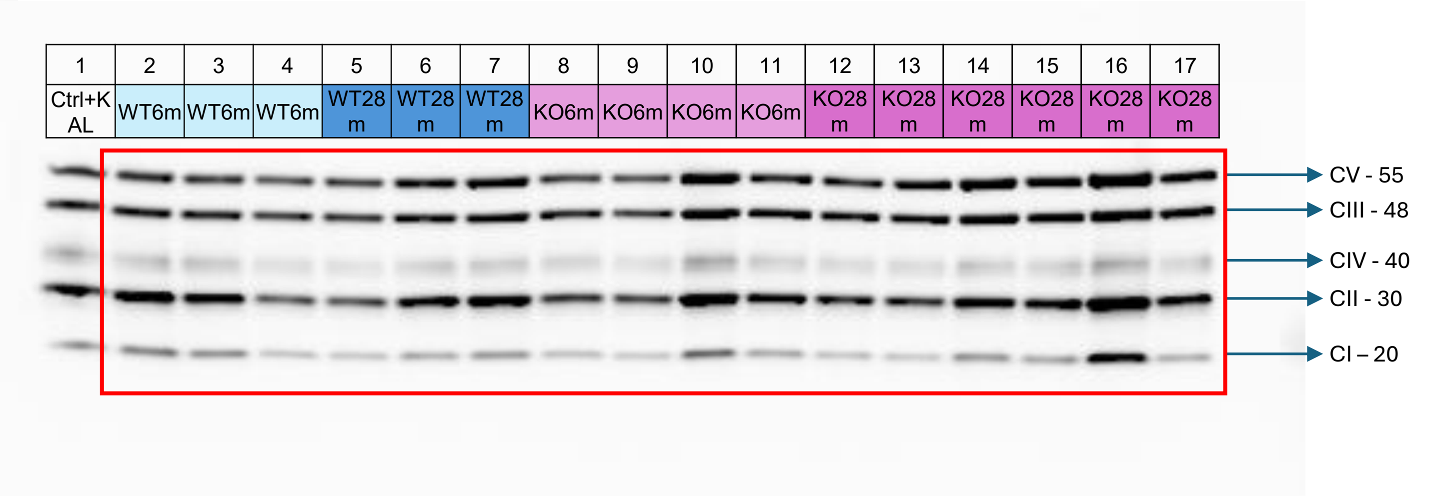

Figure 4C

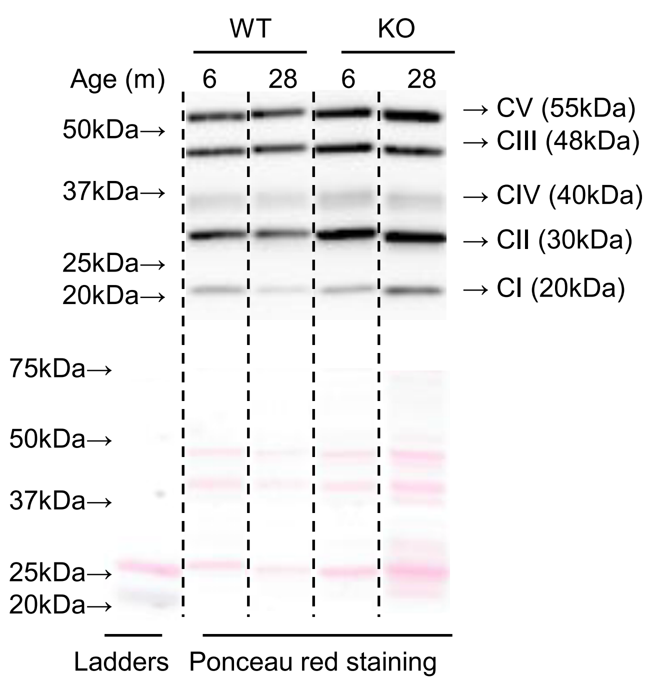

Representative blots

Used for data analysis

Full unedited blots (Figure 4G,H – data; Figure 4I – representative image)
PGC1a and p62 blot1 and 2

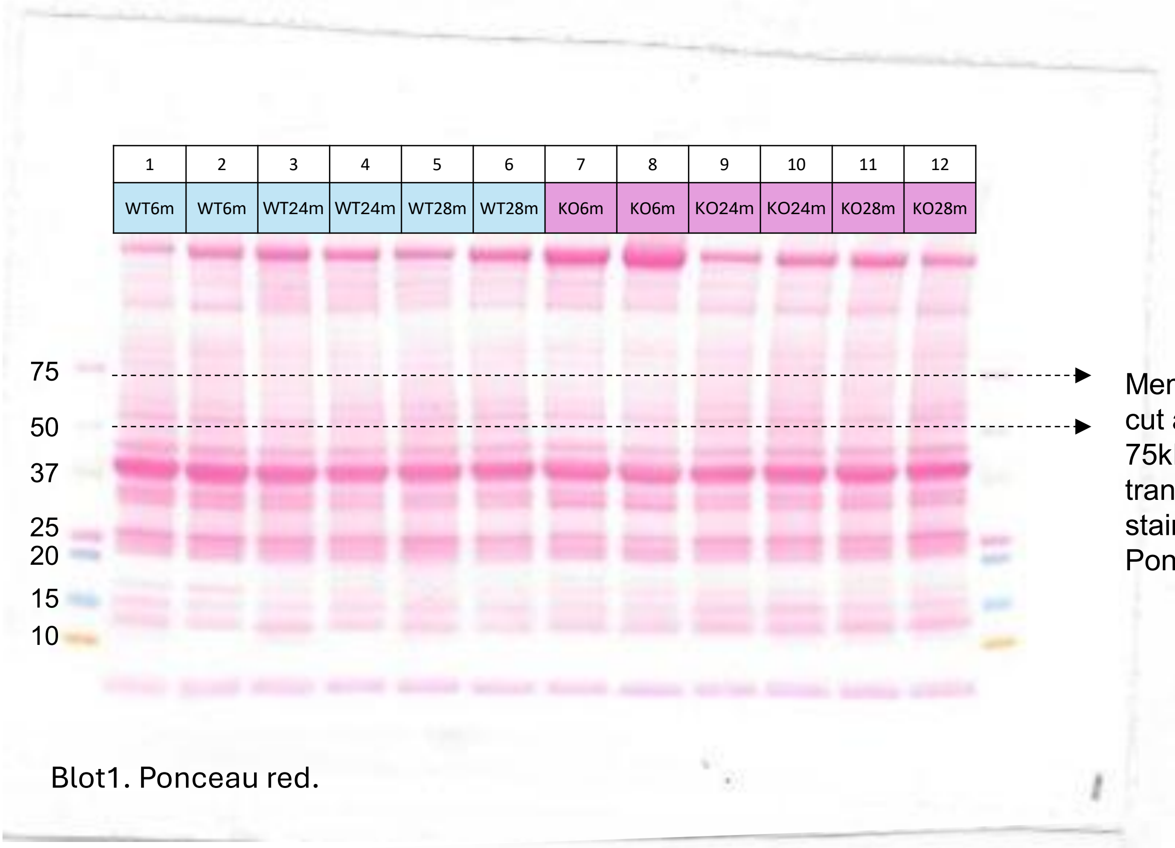

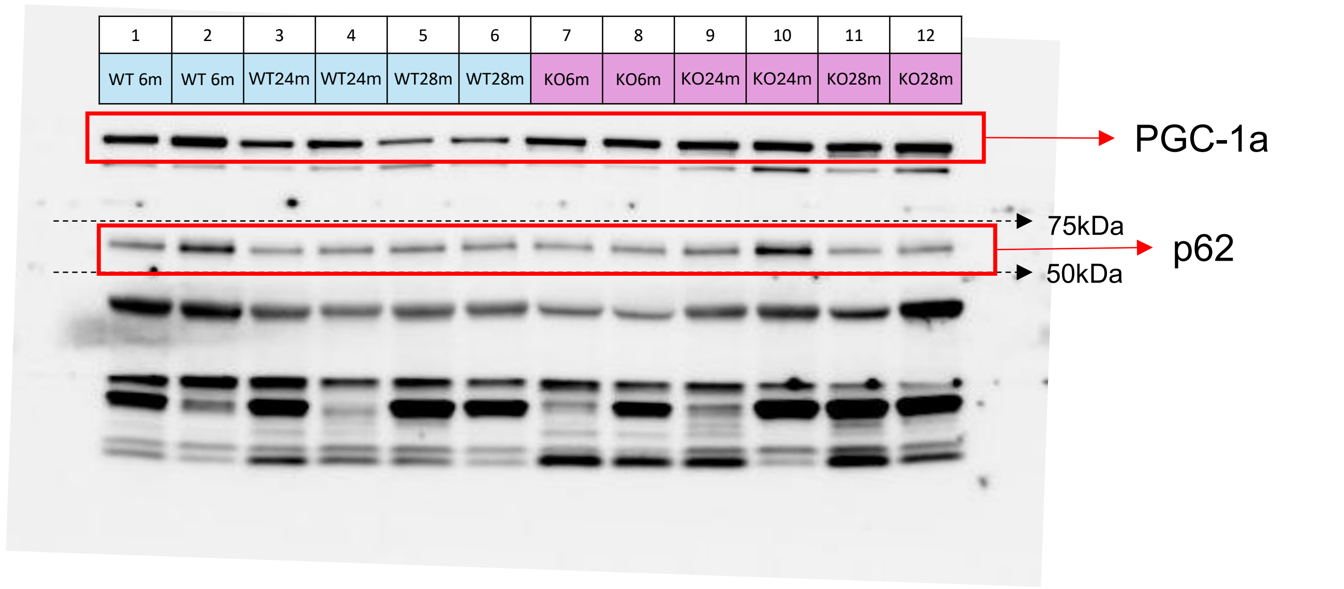

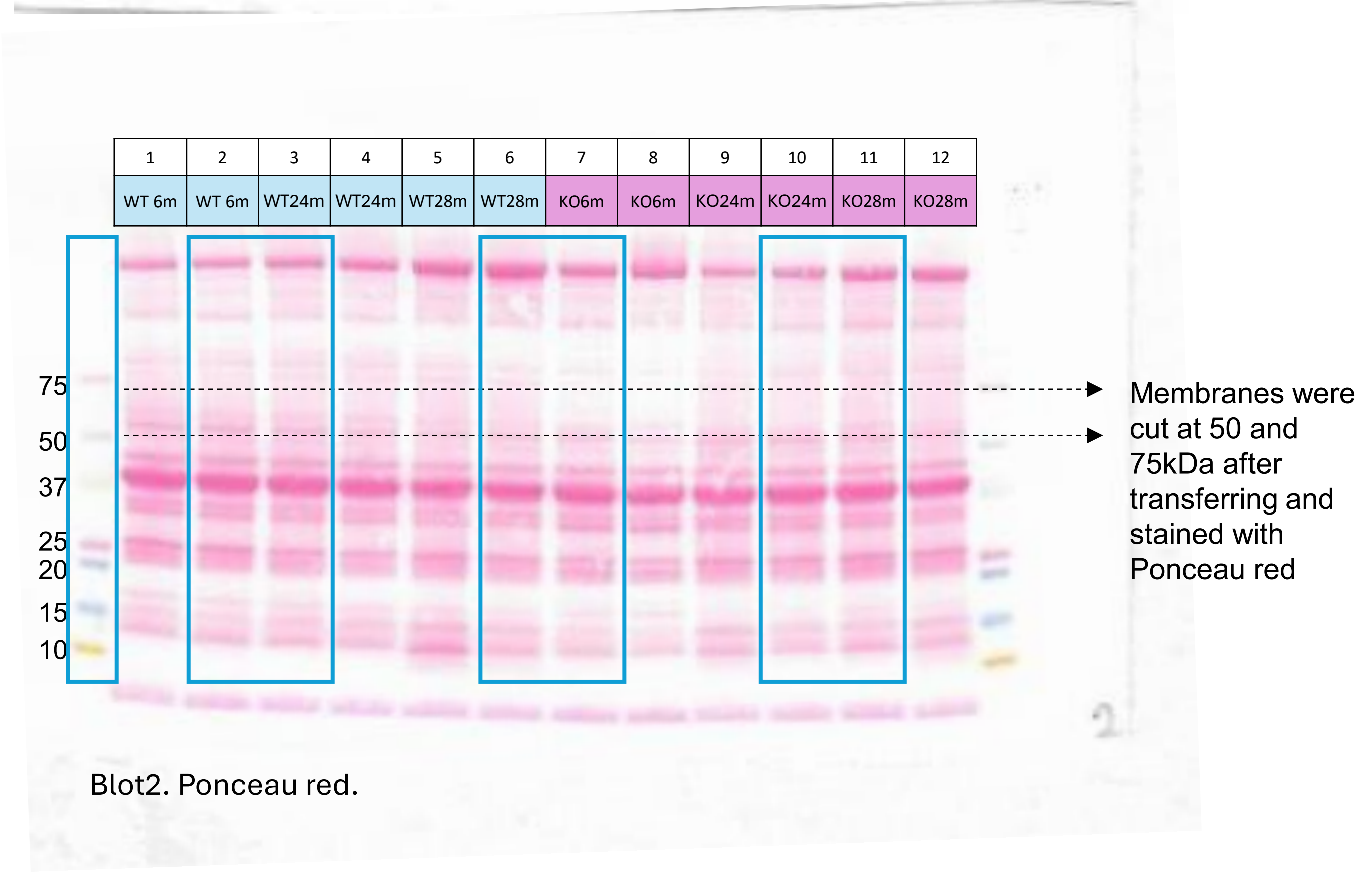

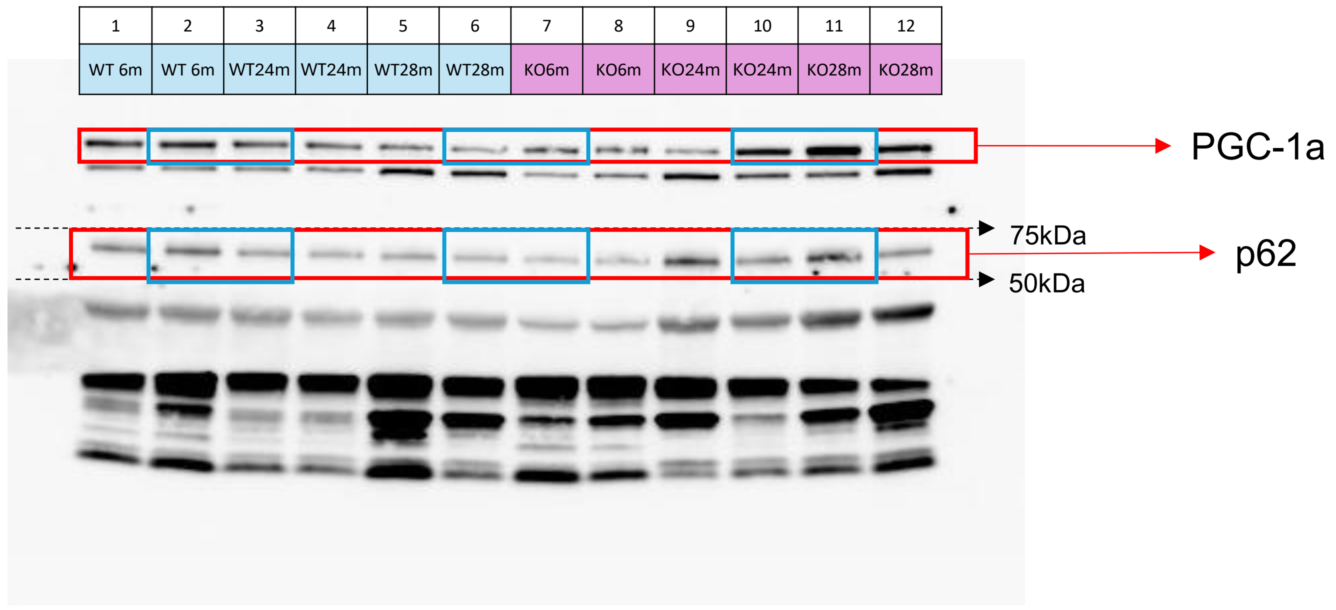

Figure 4I

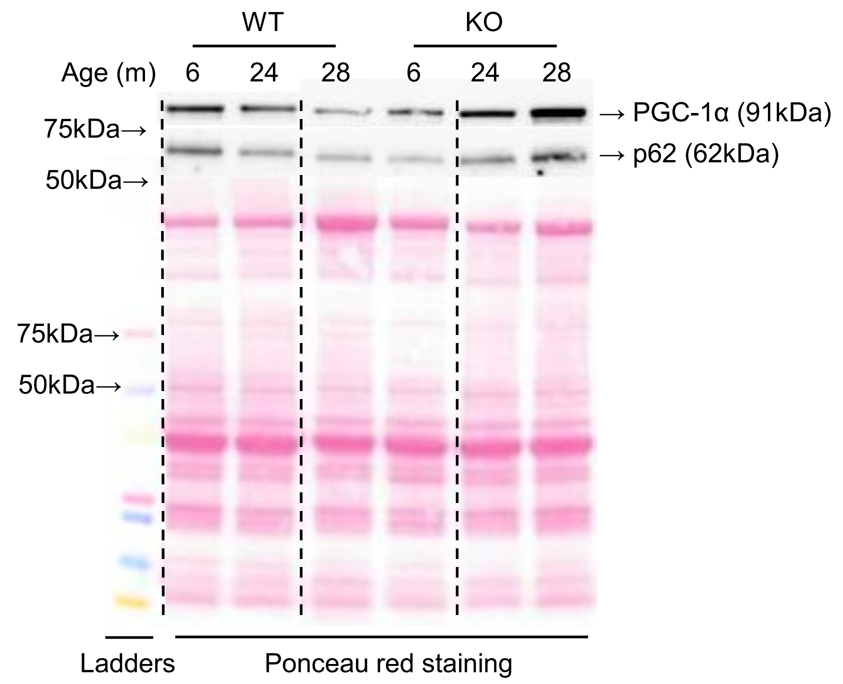

Representative blots

Used for data analysis

Full unedited blots (Figure 4J – Parkin/VDAC1)
Parkin and VDAC1– from the same blots, cut at 75, 37, and 25kDa, exposed at different times
PVDF blots (no ponceau red images)

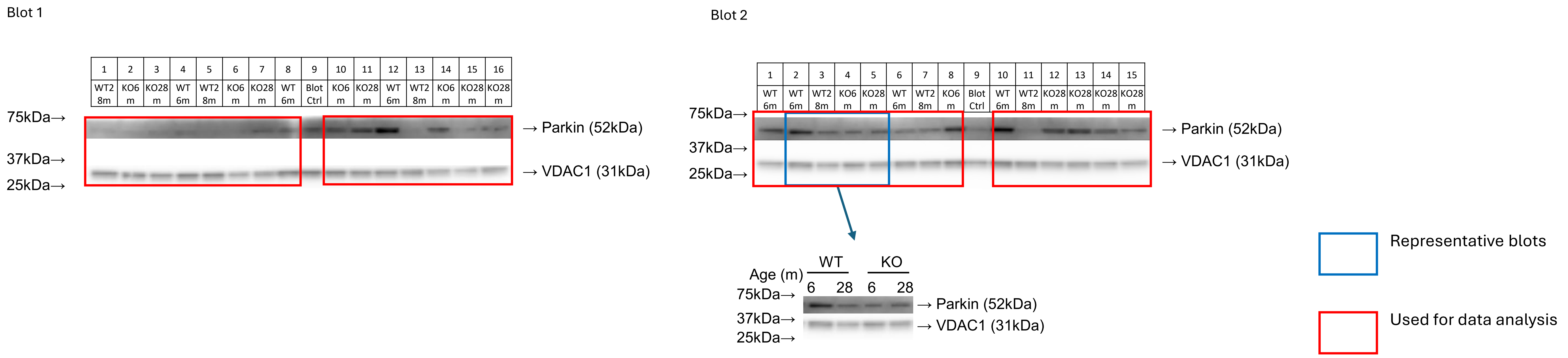

Full unedited blots (Figure 6H – data; Figure 6J– representative image)
PGC1a blot1 and 2

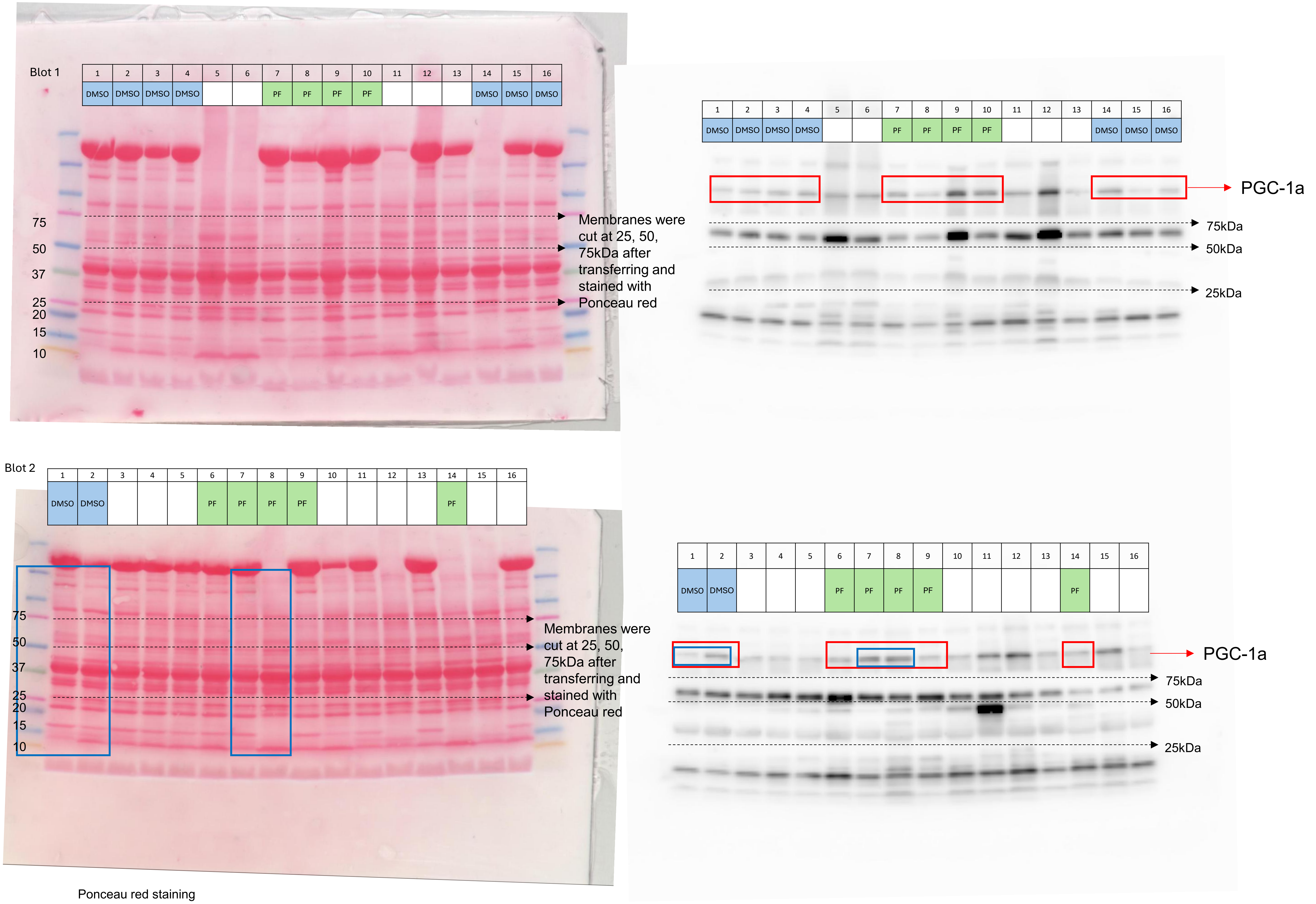

Ponceau red staining
Blanks: samples from other experiment (not included in this study)

Figure 6J

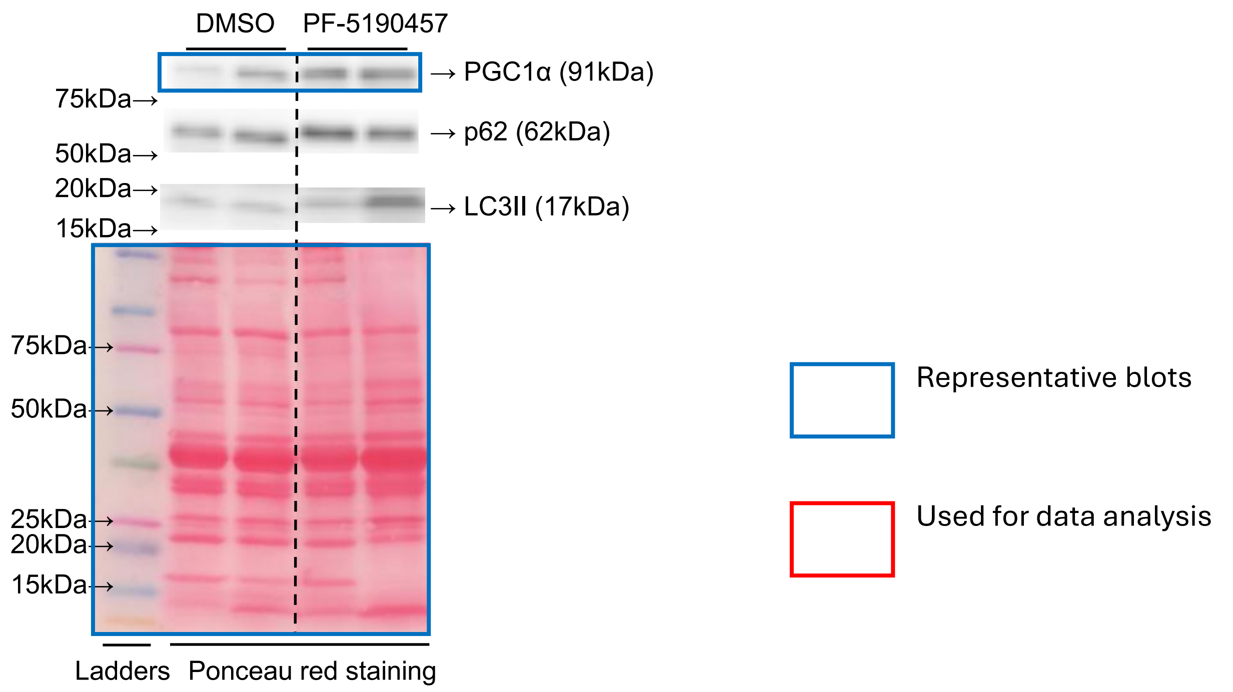

Full unedited blots (Figure 6I – data; Figure 6J– representative image)
p62 blot1 and 2 – from the same blots as PGC1a, exposed at different time

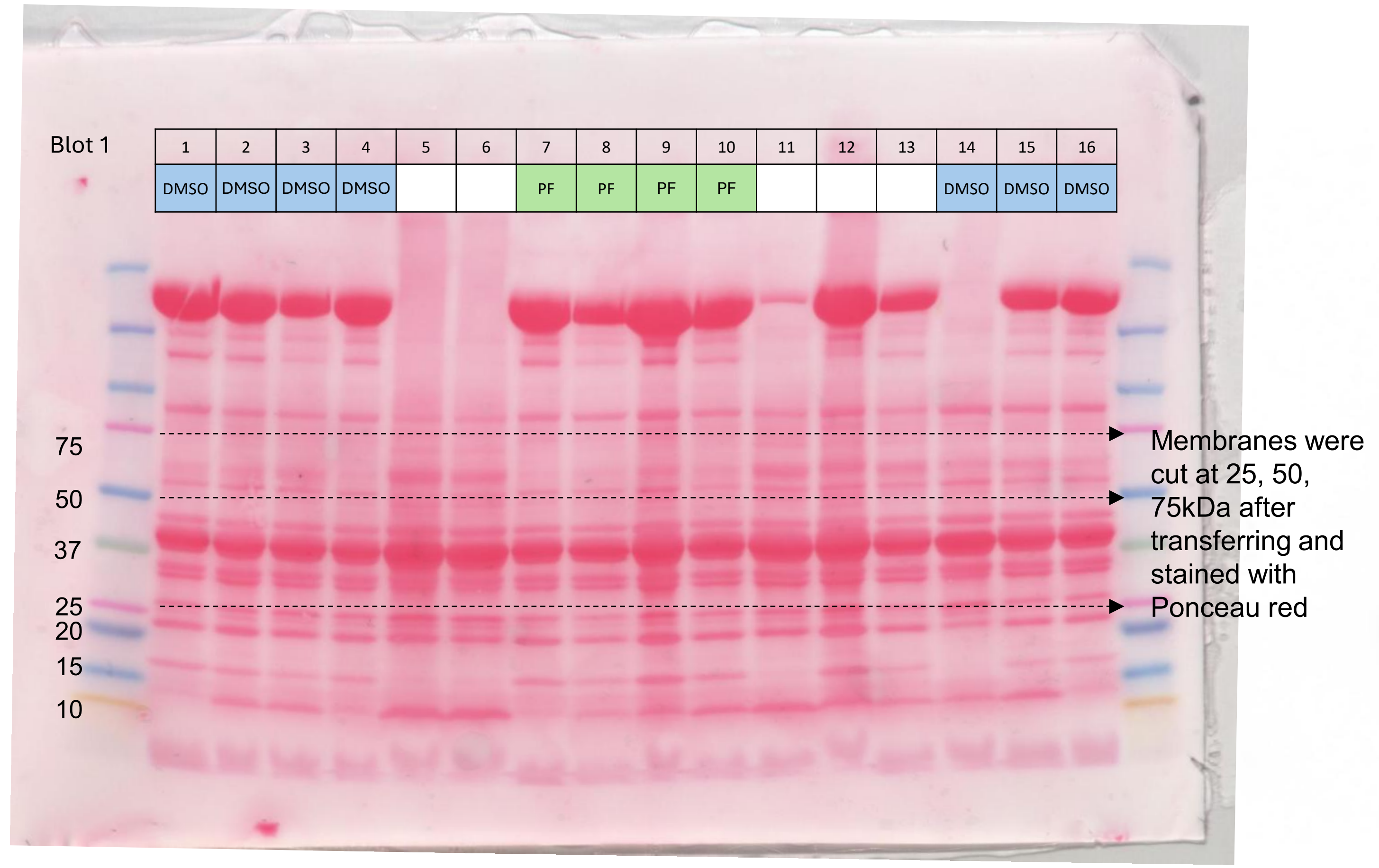

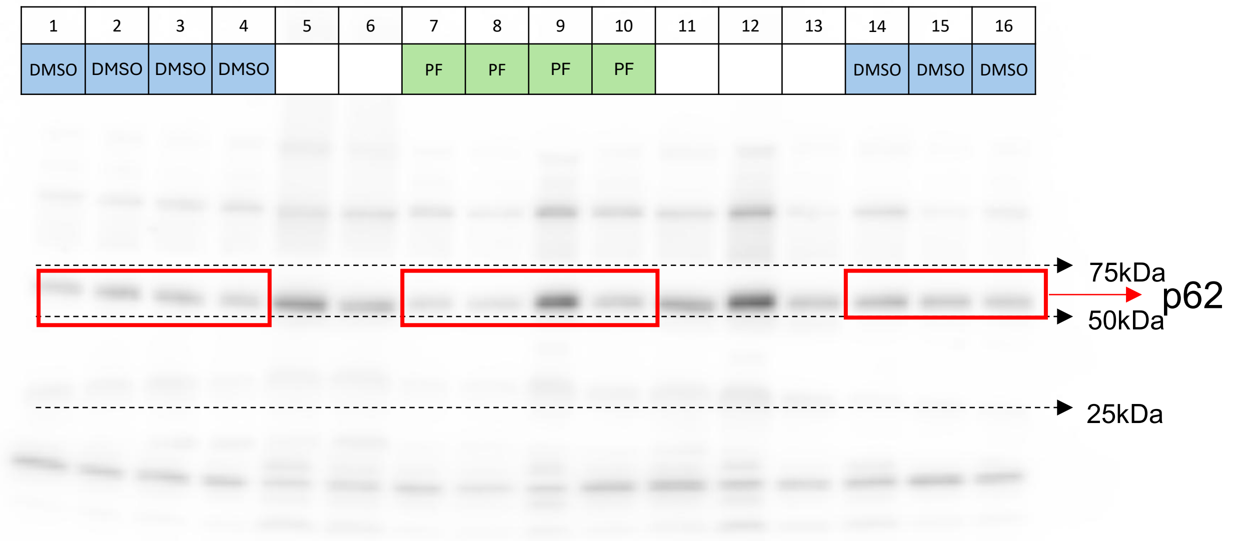

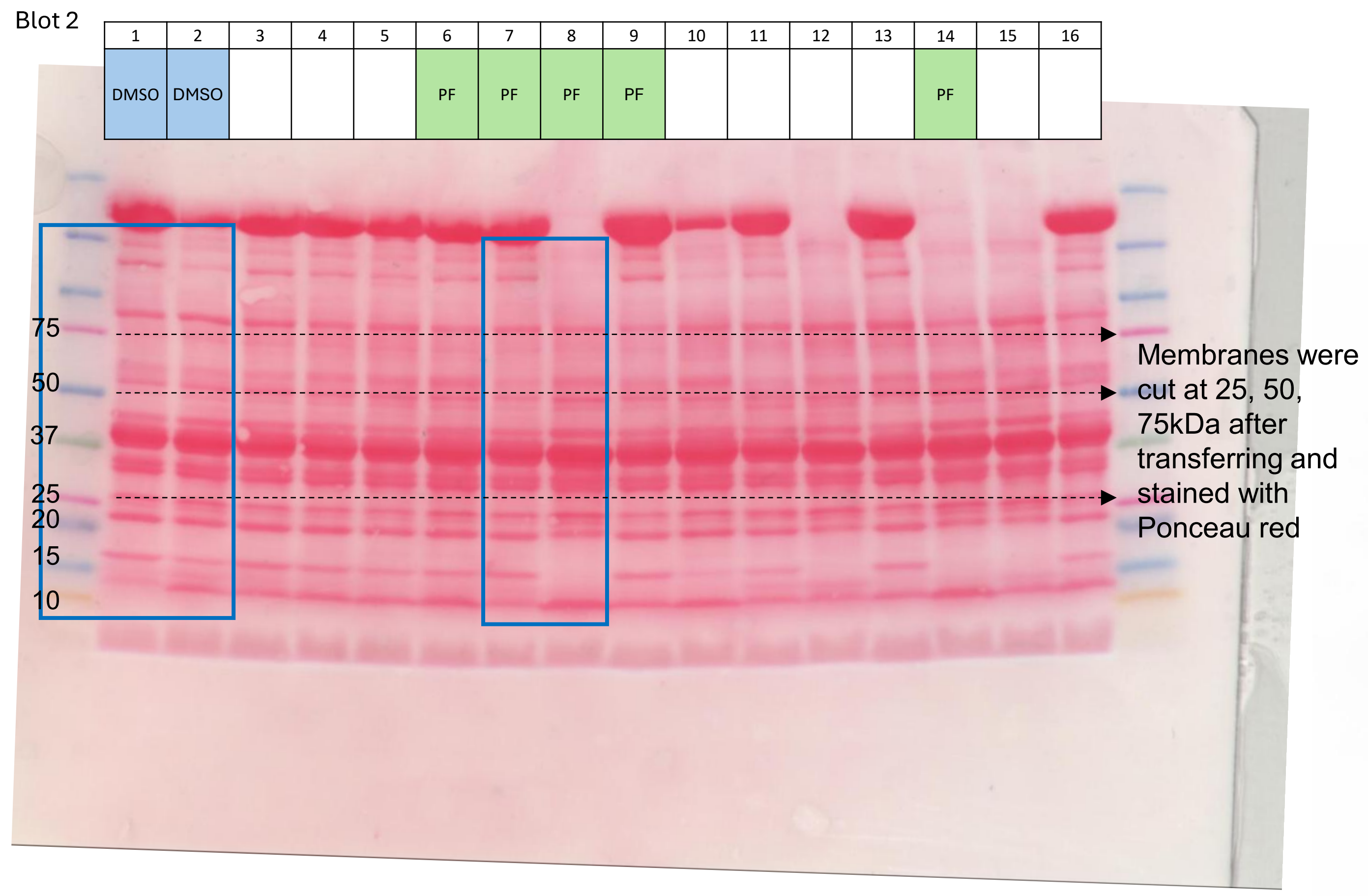

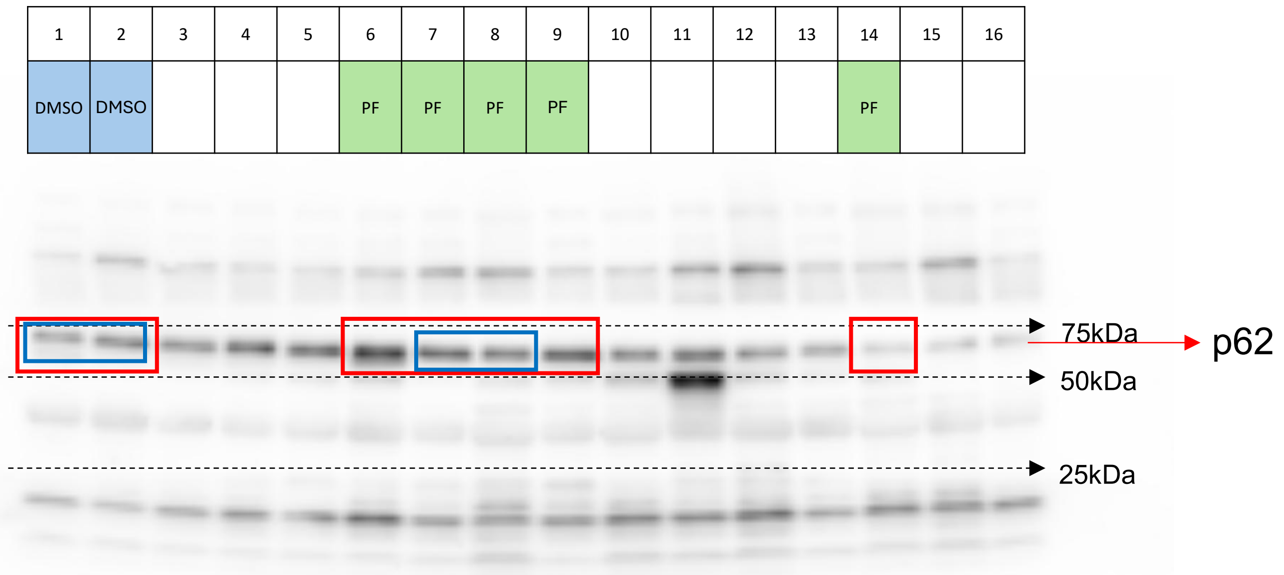

Ponceau red staining
Blanks: samples from other experiment (not included in this study)

Figure 6J

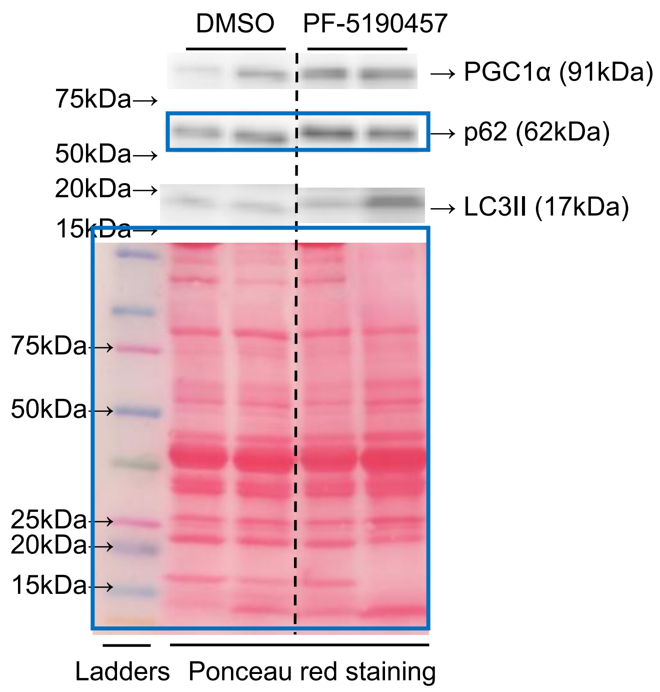

Representative blots

Used for data analysis

Full unedited blots (Figure 6I – data; Figure 6J– representative image)
LC3II blot1 and 2 – from the same blots as PGC1a, exposed at different time

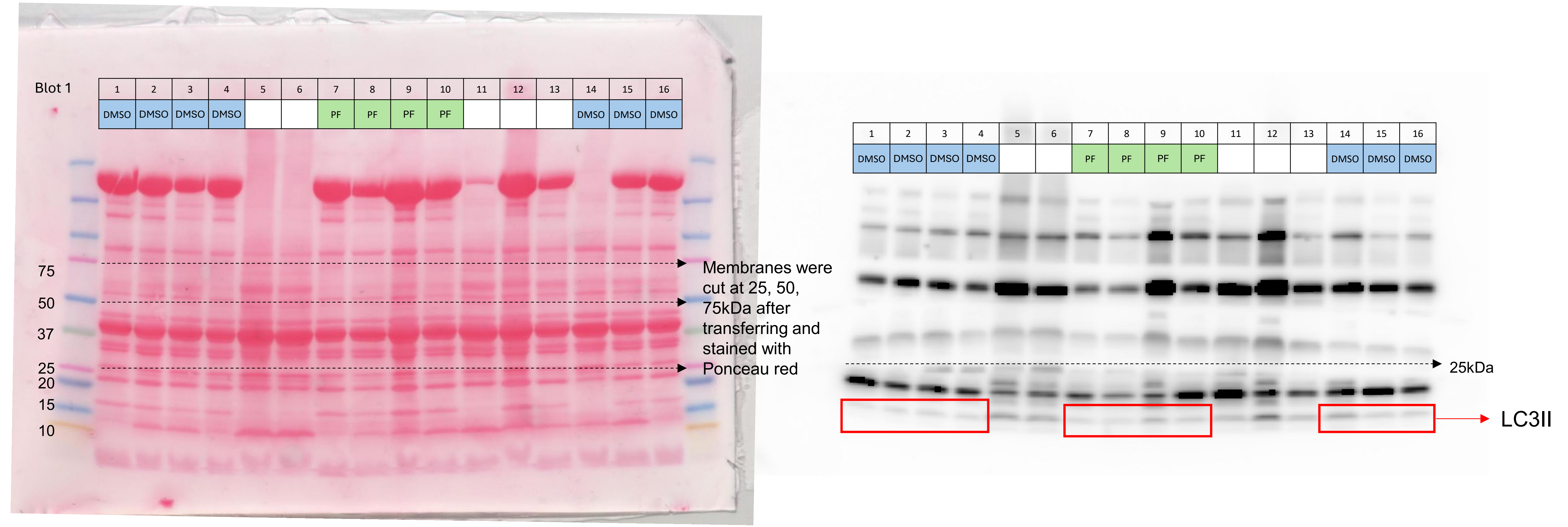

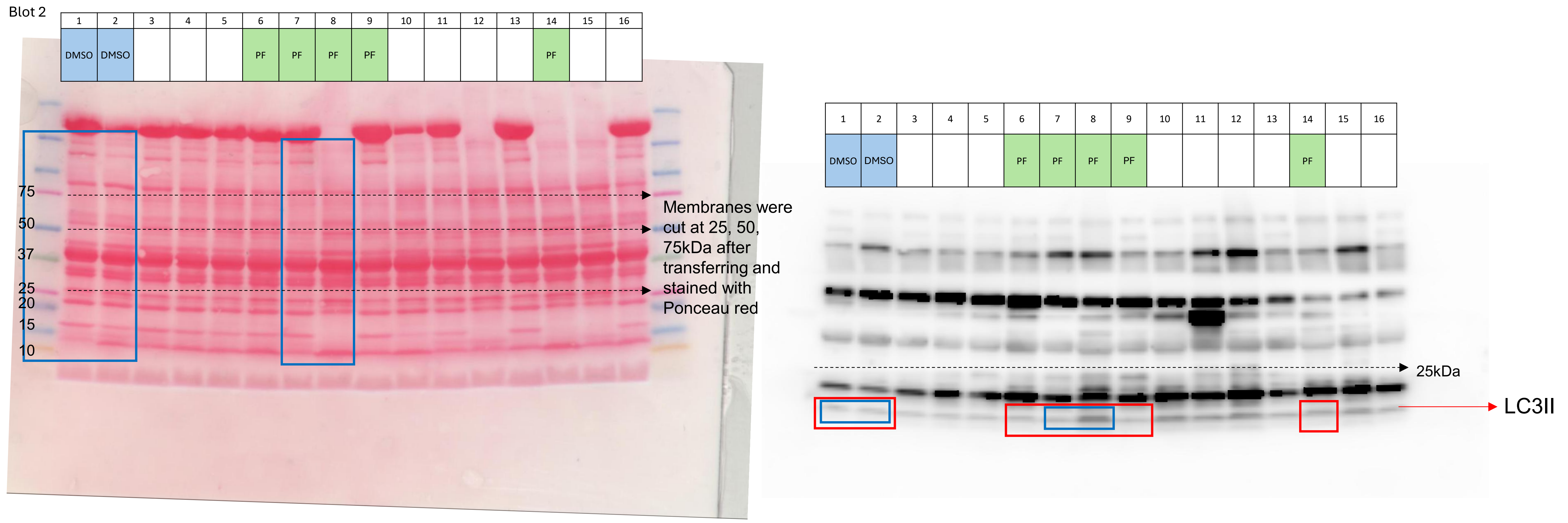

Ponceau red staining
Blanks: samples from other experiment (not included in this study)

Figure 6J

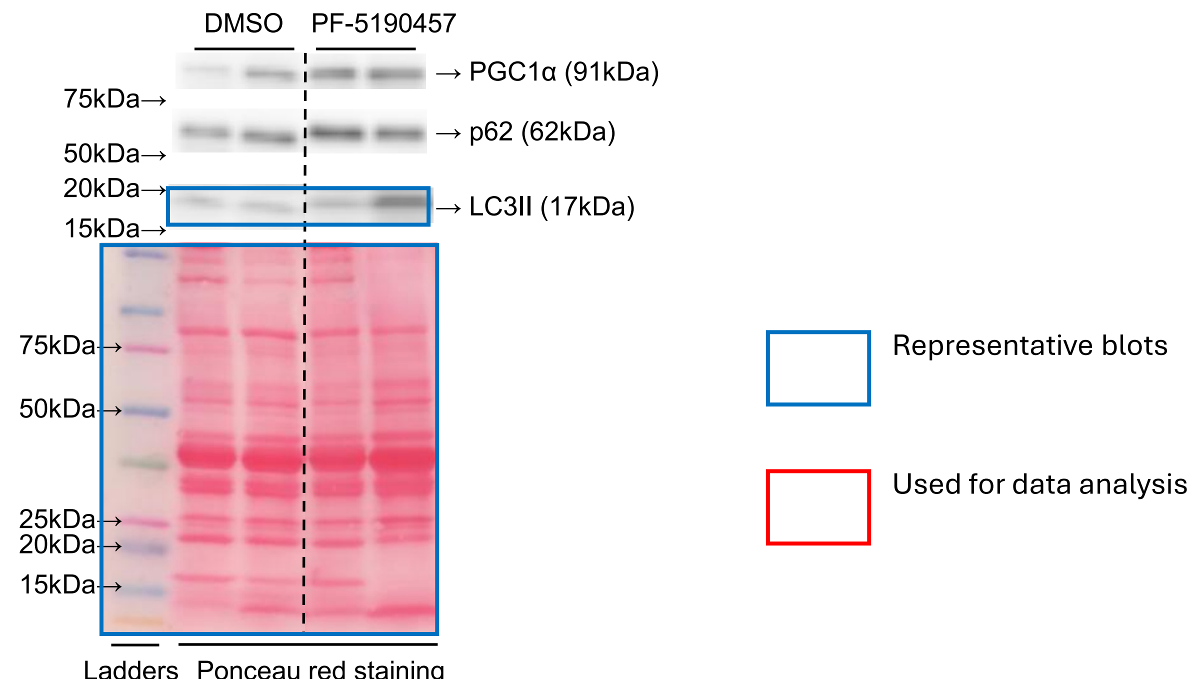

Full unedited blots (Supplemental Figure 2)
Cytochrome C blot1 and 2

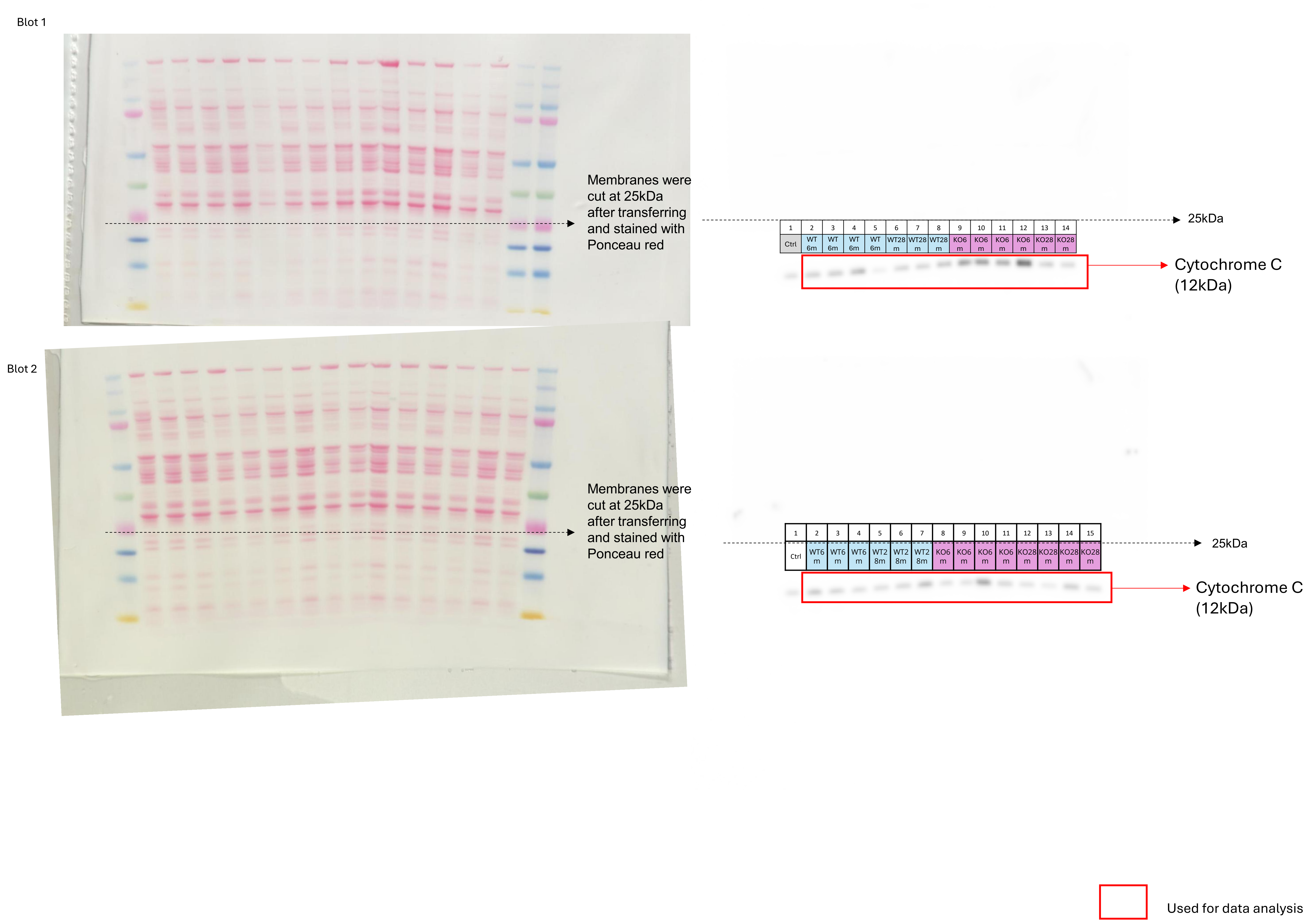

Supplement: Supplementary file 1 — Data S1: WB—GHSR aging paper FINAL. [file ACEL-25-e70472-s001.pdf]
