# Supplementary material for: Ghrelin Receptor Deletion or Pharmacological Inhibition Improves Muscle Function in Aging Male Mice
Source: Aging Cell. 2026 Apr 15;25(4):e70472. doi: 10.1111/acel.70472 (PMC13083231; doi:10.1111/acel.70472)
Supplement: Supplementary file 2 — Figure S1: GHSR‐1a deletion did not affect water content. Body free water content was evaluated using NMR in 6‐ and 28‐month‐old male GHSR‐1a wild‐type (WT) and knockout (KO) mice. ***difference between age groups with the same genotype (p < 0.001). Data are shown as mean ± SE. Sample sizes: N = 21, 11, 21, 40 (left to right bars). Figure S2: (A) Percent change (Δ, old vs. young) in each mitochondrial respiration state. $ indicates a significant difference between genotypes (p < 0.05). N = 11 (WT) and 20 (KO). (B) Cytochrome C levels in isolated mitochondria from 6‐ and 28‐month‐old male GHSR‐1a WT and KO mice. $ indicates a genotype difference within the same age group (p < 0.05). * indicates an age difference within the same genotype (p < 0.05). Data are shown as mean ± SE. Sample size: N = 6–8. Figure S3: Collagen content in TA muscles from 6‐ and 28‐month‐old male GHSR‐1a wild‐type (WT) and knockout (KO) mice evaluated by Masson's trichrome staining. (A) Quantification of collagen‐positive area is expressed as a percentage of the whole muscle cross‐sectional area. *age difference between the same genotype (p < 0.05). Data are shown as mean ± SE. Sample sizes: N = 4 for 6 m groups and N = 11–12 for 28 m groups. (B) Representative images of Masson's trichrome staining (collagen area was stained in blue). (C) Spearman's correlation was used to assess the correlation between collagen‐positive area and each sarcopenic measurement (grip strength, treadmill running time, or muscle mass). Figure S4: GHSR‐1a deletion did not affect survival. Weeks of survival in GHSR‐1a WT and KO male mice were assessed by Kaplan–Meier survival analysis (sample sizes are indicated in parentheses within legend). Differences between genotypes were analyzed using the Log‐rank (Mantel‐Cox) test (p < 0.05). N = 267 and 222 for WT and KO, respectively. Figure S5: Effects of PF‐5190457 on food intake, body composition, muscle mass and function, and mitochondrial respiration in aged mice. 25–27‐ [file ACEL-25-e70472-s002.pdf]

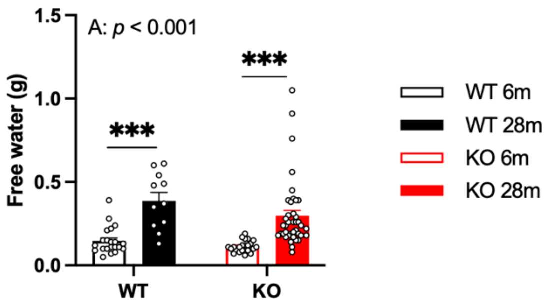

Supplemental Figure 1. GHSR-1a deletion did not affect water content. Body free water content was evaluated using NMR in 6- and 28-month-old male GHSR-1a wild-type (WT) and knockout (KO) mice. ***: difference between age groups with the same genotype (p < 0.001). Data are shown as mean ± SE. Sample sizes: N = 21, 11, 21, 40 (left to right bars).

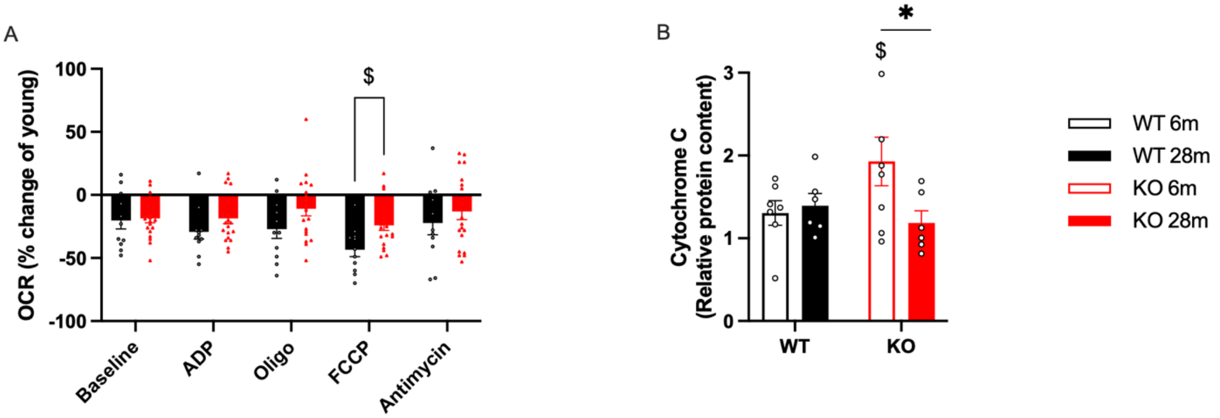

Supplemental Figure 2. (A) Percent change (Δ, old vs. young) in each mitochondrial respiration state. $ indicates a significant difference between genotypes ($p < 0.05$). N = 11 (WT) and 20 (KO). (B) Cytochrome C levels in isolated mitochondria from 6- and 28-month-old male GHSR-1a WT and KO mice. $ indicates a genotype difference within the same age group ($p < 0.05$). * indicates an age difference within the same genotype ($p < 0.05$). Data are shown as mean ± SE. Sample size: N = 6-8.

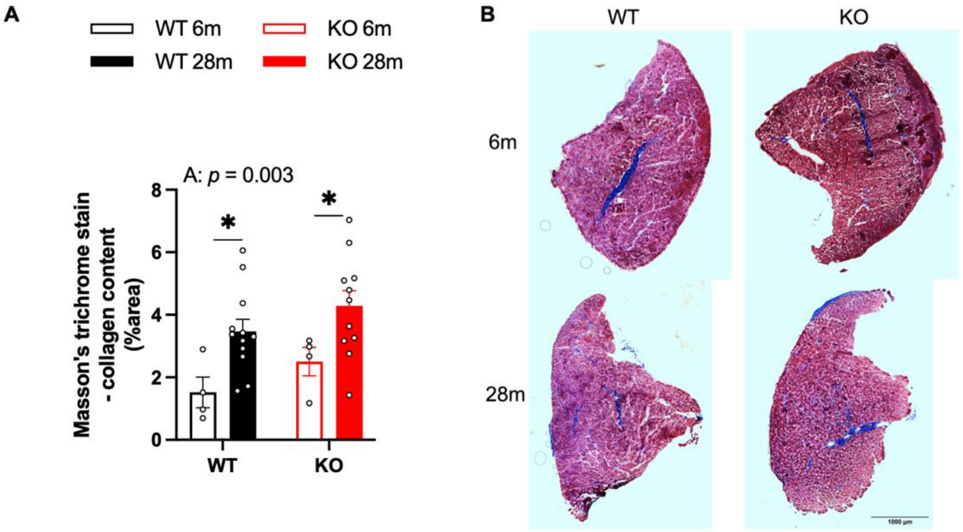

C

		Spearman's rho	Grip	Treadmill	Muscle mass
**Correlation with collagen positive area (% of total)**	Young	Correlation Coefficient	-0.79	0.07	0.29
		Sig. (2-tailed)	0.036	0.879	0.535
		N	7	7	7
	Old	Correlation Coefficient	-0.15	0.21	-0.44
		Sig. (2-tailed)	0.545	0.409	0.057
		N	19	18	19

Supplemental Figure 3. Collagen content in TA muscles from 6- and 28-month-old male GHSR-1a wild-type (WT) and knockout (KO) mice evaluated by Masson's trichrome staining. (A) Quantification of collagen-positive area is expressed as a percentage of the whole muscle cross-sectional area. *: age difference between the same genotype ($p < 0.05$). Data are shown as mean ± SE. Sample sizes: N = 4 for 6m groups and N = 11-12 for 28m groups. (B) Representative images of Masson's trichrome staining (collagen area was stained in blue). (C) Spearman's correlation was used to assess the correlation between collagen-positive area and each sarcopenic measurement (grip strength, treadmill running time, or muscle mass).

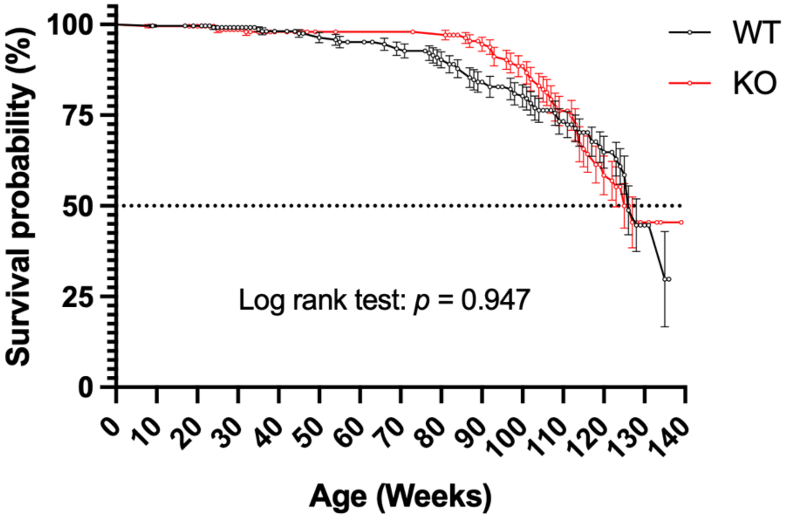

Supplemental Figure 4. GHSR-1a deletion did not affect survival. Weeks of survival in GHSR-1a WT and KO male mice were assessed by Kaplan-Meier survival analysis (sample sizes are indicated in parentheses within legend). Differences between genotypes were analyzed using the Log-rank (Mantel-Cox) test ($p < 0.05$). N = 267 and 222 for WT and KO, respectively.

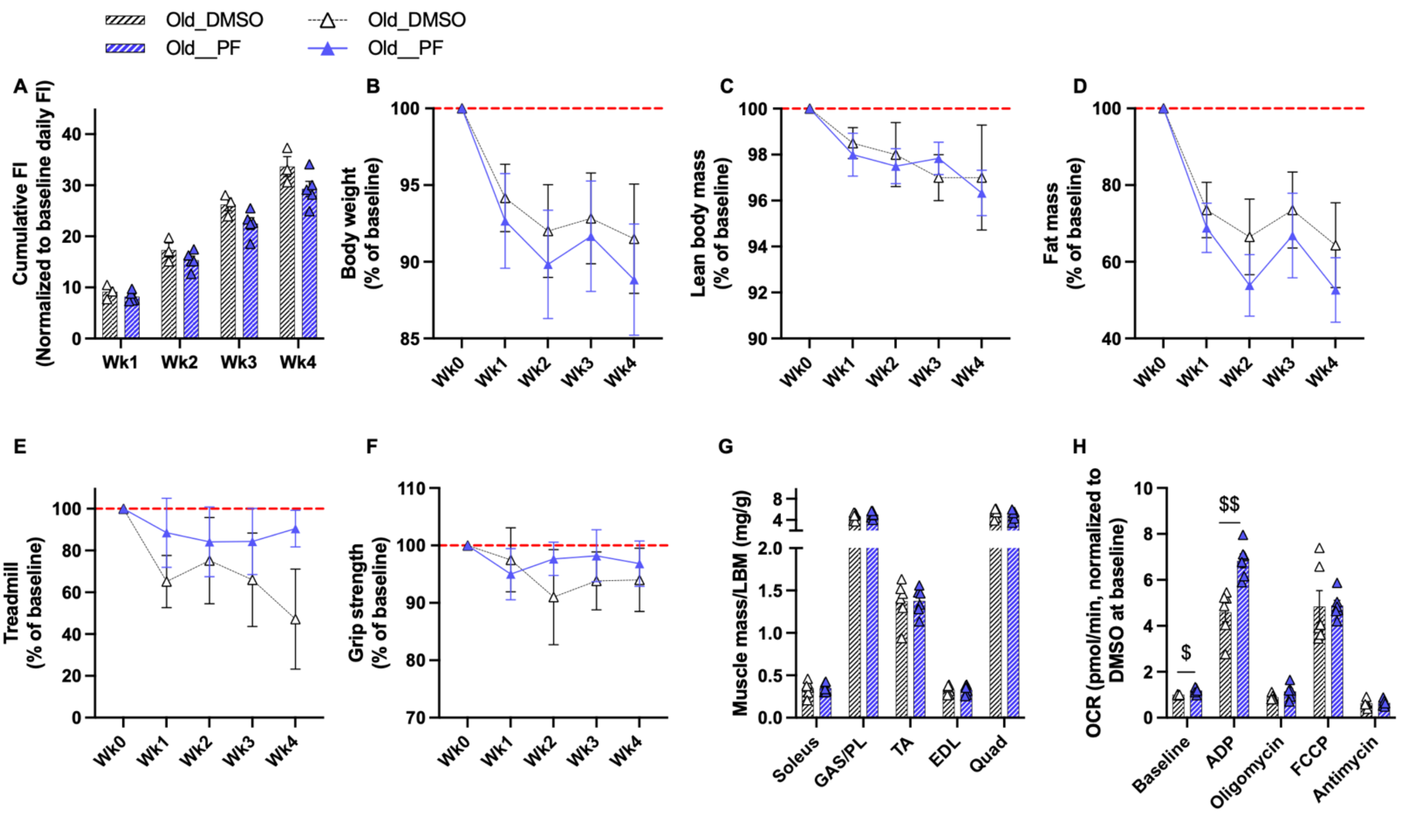

Supplemental Figure 5. Effects of PF-5190457 on food intake, body composition, muscle mass and function, and mitochondrial respiration in aged mice. 25-27-month-old C57BL/6J male mice were treated with either DMSO in saline or PF-5190457 for 28 days. Weekly measurements include (A) cumulative food intake, normalized to baseline daily food intake (g/g), (B) body weight, (C) lean body mass (LBM), (D) fat mass, (E) treadmill running time, and (F) grip strength, all expressed as a percentage of baseline levels at week 0. (G) Individual muscle mass at the end of the treatment was normalized to LBM (g/g). (H) Mitochondrial respiration, measured as the oxygen consumption rate (OCR) at different states, including baseline, and after stimulation with ADP, oligomycin, FCCP, and antimycin, in isolated mitochondria from PL muscles (pmol/min). Relative OCR levels were normalized to the baseline state in the DMSO group. Independent t-tests (two-tailed) were performed to detect differences between treatments ($, $$: $p < 0.05$ and 0.01). Trend differences were detected at $0.05 \leq p < 0.1$ and are shown in the graph. Data are presented as mean ± SE. Sample sizes: N = 3 (DMSO) and N = 5 (PF-5190457) for panel A, N = 6 for other panels.

Supplemental Table 1. Kyoto Encyclopedia of Genes and Genomes (KEGG) Pathways Correlated with Each Sarcopenia Measure Significantly Perturbed by GHSR-1a Deletion and Aging

**Treadmill**				**Top-ranked proteins**				
Pathway	N Genes	P Value	FDR	1	2	3	4	5
Complement and coagulation cascades	42	<0.0001	0.0002	IC1	CO8G	C1QB	C1QC	DAF2/DAF1
Metabolism of xenobiotics by cytochrome P450	23	<0.0001	0.004	GSTM5	CBR2	GSTA3	GSTM4	MGST3
Diabetic cardiomyopathy	123	0.0001	0.0072	NDUB9	NDUB8	NDUA8	NDUAB	NDUS7
Hepatocellular carcinoma	35	0.0006	0.0385	GSTM5	MP2K1	MP2K2	GSTA3	GSTM4
Thermogenesis	108	0.0008	0.0431	NDUB9	NDUB8	NDUA8	NDUAB	MGLL

**Grip**				**Top-ranked proteins**				
Pathway	N Genes	P Value	FDR	1	2	3	4	5
Complement and coagulation cascades	42	<0.0001	0.0027	E9Q6D8	CO4B	C1QB	C1QC	THRB
Valine, leucine and isoleucine degradation	37	0.0002	0.0243	ODBA	IVD	HCD2	PCCA	MCCA
Propanoate metabolism	27	0.0003	0.0243	DCMC	ODBA	PCCA	LDHB	ACADS
Chemical carcinogenesis - DNA adducts	19	0.0005	0.0355	GSTK1	GSTA4	GSTT1	CP2E1	ST1A1

**Muscle**				**Top-ranked proteins**				
Pathway	N Genes	P Value	FDR	1	2	3	4	5
Complement and coagulation cascades	42	<0.0001	<0.0001	KLKB1	THRB	PLMN	A2AP	PROC
Neuroactive ligand-receptor interaction	11	<0.0001	0.0001	THRB	PLMN	ANGT	KNG1	CO5
Oxidative phosphorylation	83	<0.0001	0.0002	NDUB4	NDUB2	COX6C	ATP5L	CYC
Chemical carcinogenesis - reactive oxygen species	114	<0.0001	0.0008	NDUB4	NDUB2	COX6C	ADT1	ADT2
Citrate cycle (TCA cycle)	26	<0.0001	0.0012	CISY	IDHC	SUCB1	IDHG1	ODO2
Non-alcoholic fatty liver disease	81	0.0001	0.0023	NDUB4	NDUB2	COX6C	CYC	NDUA6
Systemic lupus erythematosus	25	0.0001	0.0027	E9Q6D8	CO8G	CO8A	CO5	C1QB
2-Oxocarboxylic acid metabolism	24	0.0001	0.0029	AATM	CISY	AATC	IDHC	IDHG1
Diabetic cardiomyopathy	123	0.0001	0.0029	ANGT	NDUB4	KCC2B	NDUB2	COX6C
Staphylococcus aureus infection	27	0.0002	0.0037	PLMN	CAMP	FIBG	CO5	C1QB
Propanoate metabolism	27	0.0002	0.0037	ECHD1	LDHB	HIBCH	DCMC	SUCB1
Thermogenesis	108	0.0002	0.0037	PLIN1	NDUB4	NDUF6	NDUB2	COX6C
Alzheimer disease	172	0.0003	0.0062	NDUB4	NDUB2	COX6C	PPID	ADT1
Parkinson disease	152	0.0003	0.0062	NDUB4	KCC2B	NDUB2	COX6C	ADT1

Alanine, aspartate and glutamate metabolism	19	0.0005	0.0086	AATM	SSDH	AATC	ASGL1	PURA2
Valine, leucine and isoleucine degradation	37	0.0007	0.0113	SCOT1	IVD	HIBCH	HCD2	ACADS
Metabolic pathways	532	0.0009	0.0133	MECR	PHOP1	AOC3	SCOT1	AATM
Huntington disease	154	0.0011	0.0157	NDUB4	NDUB2	COX6C	ADT1	PSD13
Amyotrophic lateral sclerosis	179	0.0016	0.0225	NDUB4	NDUB2	COX6C	PSD13	CYC
Regulation of lipolysis in adipocytes	14	0.0017	0.0225	PLIN1	ABHD5	MGLL	P85A	LIPS
Pathways of neurodegeneration - multiple diseases	214	0.0019	0.0244	NDUB4	KCC2B	NDUB2	COX6C	PPID
Carbon metabolism	85	0.0027	0.0321	AATM	CISY	HIBCH	AATC	ALDOC
Lipoic acid metabolism	10	0.0034	0.0391	ODO2	DLDH	ODBB	ODPA	ODBA
Prion disease	165	0.0039	0.0417	E9Q6D8	CO8G	CO8A	NDUB4	CO5
ECM-receptor interaction	33	0.0038	0.0417	FINC	TSP1	ITB3	CO1A1	VTNC

Interaction terms between age and genotype were analyzed to identify KEGG pathways correlated with each sarcopenia measure (treadmill, grip, and muscle mass) that were significantly influenced by GHSR-1a deletion (WT vs. KO) and age (6 months vs. 28 months, FDR < 0.05). The top 5 ranked proteins from each pathway are listed. FDR: False Discovery Rate.

Supplemental Table 2. Gene Ontology Cellular Component Categories for Top 5 Proteins Detected in Interaction Term.

Category	Gene Ontology (Cellular Component)	Proteins
Cytoplasm	axon [GO:0030424]	CO4B; MGLL; MP2K1
Cytoplasm	axon terminus [GO:0043679]	AATC
Cytoplasm	cell cortex [GO:0005938]	FIBG; MP2K1
Cytoplasm	cell projection [GO:0042995]	CAMP
Cytoplasm	centrosome [GO:0005813]	KCC2B; MP2K1
Cytoplasm	cytoplasm [GO:0005737]	ASGL1; CO1A1; DCMC; LIPS; MP2K2; P85A; PPID
Cytoplasm	cytosol [GO:0005829]	AATC; ABHD5; CYC; ECHD1; GSTA3; GSTM4; GSTM5; GSTT1; IDHC; KCC2B; LDHB; LIPS; MECR; MGLL; MP2K1; MP2K2; ODO2; P85A; PLIN1; PPID; PURA2; RASN/RASH; ST1A1
Cytoplasm	dendrite [GO:0030425]	CO4B
Cytoplasm	dendrite cytoplasm [GO:0032839]	MP2K1
Cytoplasm	microtubule [GO:0005874]	MP2K1; MP2K2
Cytoplasm	microvillus [GO:0005902]	AOC3
Cytoplasm	myelin sheath [GO:0043209]	AATM; ADT1; ADT2; CYC; DLDH; LDHB; ODO2; ODPA; SUCB1
Cytoplasm	perikaryon [GO:0043204]	MP2K1
Cytoplasm	perinuclear region of cytoplasm [GO:0048471]	MP2K1; MP2K2; P85A
Cytoplasm	sperm fibrous sheath [GO:0035686]	GSTM5
Cytoplasm	spindle midzone [GO:0051233]	KCC2B
Cytoplasm	varicosity [GO:0043196]	MGLL
Cytoplasm	early endosome [GO:0005769]	AOC3; MP2K1; MP2K2
Cytoplasm	late endosome [GO:0005770]	MP2K1; MP2K2
Cytoplasm	lipid droplet [GO:0005811]	ABHD5; LIPS; MGLL; PLIN1
Cytoplasm	melanosome [GO:0042470]	ITB3
Cytoplasm	peroxisomal matrix [GO:0005782]	DCMC; IDHC
Cytoplasm	peroxisome [GO:0005777]	DCMC; GSTK1
Cytoplasm	secretory granule [GO:0030141]	CO1A1
Cytoplasm	specific granule [GO:0042581]	CAMP
Cytoplasm	apoptosome [GO:0043293]	CYC
Cytoplasm	catalytic complex [GO:1902494]	ODPA; PCCA
Cytoplasm	GTPase complex [GO:1905360]	RASN/RASH
Cytoplasm	oxidoreductase complex [GO:1990204]	LDHB
Cytoplasm	peptidase inhibitor complex [GO:1904090]	VTNC
Cytoplasm	phosphatidylinositol 3-kinase complex [GO:0005942]	P85A
Cytoplasm	phosphatidylinositol 3-kinase complex, class IA [GO:0005943]	P85A
Cytoplasm	acrosomal matrix [GO:0043159]	DLDH
Endoplasmic Reticulum	endoplasmic reticulum [GO:0005783]	AOC3; CP2E1; HCD2; MP2K1; MP2K2; PLIN1; PROC; TSP1

Endoplasmic Reticulum	endoplasmic reticulum membrane [GO:0005789]	CP2E1
Endoplasmic Reticulum	perinuclear endoplasmic reticulum membrane [GO:1990578]	P85A
Endoplasmic Reticulum	rough endoplasmic reticulum lumen [GO:0048237]	VTNC
Endoplasmic Reticulum	sarcoplasmic reticulum [GO:0016529]	TSP1
Endoplasmic Reticulum	sarcoplasmic reticulum membrane [GO:0033017]	KCC2B
Extracellular Region	basement membrane [GO:0005604]	FINC; VTNC
Extracellular Region	collagen trimer [GO:0005581]	C1QB; C1QC; CO1A1
Extracellular Region	collagen type I trimer [GO:0005584]	CO1A1
Extracellular Region	collagen-containing extracellular matrix [GO:0062023]	A2AP; CO1A1; FIBG; FINC; IC1; KNG1; PLMN; THRB; TSP1; VTNC
Extracellular Region	extracellular exosome [GO:0070062]	FINC
Extracellular Region	extracellular matrix [GO:0031012]	CO1A1; FINC; PHOP1; TSP1; VTNC
Extracellular Region	extracellular membrane-bounded organelle [GO:0065010]	PHOP1
Extracellular Region	extracellular region [GO:0005576]	ANGT; C1QB; C1QC; CO1A1; CO8G; E9Q6D8; KNG1; PLMN; PROC; TSP1
Extracellular Region	extracellular space [GO:0005615]	ANGT; C1QB; C1QC; CAMP; CO1A1; CO4B; CO5; CO8A; FINC; IC1; KLKB1; PLMN; THRB; TSP1; VTNC
Extracellular Region	fibrinogen complex [GO:0005577]	A2AP; FIBG; FINC
Extracellular Region	complement component C1 complex [GO:0005602]	C1QB; C1QC
Extracellular Region	complement component C1q complex [GO:0062167]	C1QB; C1QC
Extracellular Region	membrane attack complex [GO:0005579]	CO5; CO8A; CO8G; E9Q6D8
Golgi Apparatus	cis-Golgi network [GO:0005801]	P85A
Golgi Apparatus	endoplasmic reticulum-Golgi intermediate compartment [GO:0005793]	FINC
Golgi Apparatus	Golgi apparatus [GO:0005794]	AOC3; MP2K1; MP2K2; PROC; RASN/RASH
Golgi Apparatus	Golgi lumen [GO:0005796]	VTNC
Golgi Apparatus	Golgi membrane [GO:0000139]	RASN/RASH
Mitochondrion	mitochondrial inner membrane [GO:0005743]	AATM; ADT1; ADT2; ATP5L; COX6C; CP2E1; GSTK1; HCD2; LDHB; MCCA; NDUA6; NDUA8; NDUAB; NDUB2; NDUB4; NDUB8; NDUB9; NDUF6; NDUS7; ODBB
Mitochondrion	mitochondrial intermembrane space [GO:0005758]	CYC; NDUA8

Mitochondrion	mitochondrial matrix [GO:0005759]	AATM; ACADS; CBR2; CISY; DCMC; DLDH; GSTK1; IVD; MCCA; ODBA; ODBB; ODO2; ODPA; PCCA
Mitochondrion	mitochondrial membrane [GO:0031966]	ADT1; IVD
Mitochondrion	mitochondrial nucleoid [GO:0042645]	ADT2; HCD2
Mitochondrion	mitochondrial outer membrane [GO:0005741]	ADT1; MGST3
Mitochondrion	mitochondrial permeability transition pore complex [GO:0005757]	ADT1; ADT2
Mitochondrion	mitochondrial ribonuclease P complex [GO:0030678]	HCD2
Mitochondrion	mitochondrion [GO:0005739]	AATM; ACADS; ADT1; ADT2; ALDOC; ATP5L; CBR2; CISY; COX6C; CYC; DCMC; DLDH; GSTA3; GSTA4; GSTK1; HCD2; HIBCH; IDHC; IDHG1; IVD; LDHB; LIPS; MCCA; MECR; MP2K1; MP2K2; NDUA6; NDUA8; NDUAB; NDUB2; NDUB4; NDUB8; NDUB9; NDUF6; NDUS7; ODBA; ODBB; ODO2; ODPA; PCCA; PURA2; SCOT1; SSDH; SUCB1
Mitochondrion	proton-transporting ATP synthase complex [GO:0045259]	ATP5L
Mitochondrion	respiratory chain complex I [GO:0045271]	NDUA6; NDUA8; NDUAB; NDUB2; NDUB4; NDUB8; NDUB9; NDUS7
Mitochondrion	respiratory chain complex IV [GO:0045277]	COX6C
Mitochondrion	branched-chain alpha-ketoacid dehydrogenase complex [GO:0160157]	DLDH; ODBA; ODBB
Mitochondrion	isocitrate dehydrogenase complex (NAD+) [GO:0045242]	IDHG1
Mitochondrion	methylcrotonoyl-CoA carboxylase complex [GO:1905202]	MCCA
Mitochondrion	oxoadipate dehydrogenase complex [GO:0160167]	DLDH; ODO2
Mitochondrion	oxoglutarate dehydrogenase complex [GO:0045252]	DLDH; ODO2
Mitochondrion	pyruvate dehydrogenase complex [GO:0045254]	DLDH; ODPA
Nucleus	nucleolus [GO:0005730]	ODBB; ODPA; PPID
Nucleus	nucleoplasm [GO:0005654]	ABHD5; ITB3; IVD; NDUB4; ODBB; ODO2; PPID; RASN/RASH
Nucleus	nucleus [GO:0005634]	DLDH; GSTT1; ITB3; LIPS; MECR; MP2K1; MP2K2; ODO2; P85A; PPID
Nucleus	acetyltransferase complex [GO:1902493]	DLDH
Nucleus	MMXD complex [GO:0071817]	ADT2
Plasma Membrane	apical plasma membrane [GO:0016324]	FINC; ITB3
Plasma Membrane	caveola [GO:0005901]	LIPS
Plasma Membrane	cytoplasmic side of plasma membrane [GO:0009898]	MP2K2
Plasma Membrane	external side of plasma membrane [GO:0009897]	DAF2/DAF1; ITB3; PLMN; THRB

Plasma Membrane	filopodium membrane [GO:0031527]	ITB3
Plasma Membrane	focal adhesion [GO:0005925]	ITB3; MP2K1; MP2K2
Plasma Membrane	lamellipodium membrane [GO:0031258]	ITB3
Plasma Membrane	membrane [GO:0016020]	AOC3; DAF2/DAF1; LIPS; MECR; MGLL; P85A; RASN/RASH
Plasma Membrane	membrane raft [GO:0045121]	LDHB
Plasma Membrane	microvillus membrane [GO:0031528]	ITB3
Plasma Membrane	plasma membrane [GO:0005886]	AATM; AOC3; CO8A; CO8G; E9Q6D8; MP2K1; RASN/RASH
Plasma Membrane	receptor complex [GO:0043235]	ITB3
Plasma Membrane	ruffle membrane [GO:0032587]	ITB3
Plasma Membrane	alpha9-beta1 integrin-ADAM8 complex [GO:0071133]	ITB3
Plasma Membrane	alphav-beta3 integrin-HMGB1 complex [GO:0035868]	ITB3
Plasma Membrane	alphav-beta3 integrin-IGF-1-IGF1R complex [GO:0035867]	ITB3
Plasma Membrane	alphav-beta3 integrin-PKCalpha complex [GO:0035866]	ITB3
Plasma Membrane	integrin alpha9-beta1 complex [GO:0034679]	ITB3
Plasma Membrane	integrin alphaIIb-beta3 complex [GO:0070442]	ITB3
Plasma Membrane	integrin alphav-beta3 complex [GO:0034683]	ITB3
Plasma Membrane	protein complex involved in cell-matrix adhesion [GO:0098637]	VTNC
Plasma Membrane	cell surface [GO:0009986]	A2AP; AOC3; ITB3; PLMN; TSP1
Plasma Membrane	cell-cell junction [GO:0005911]	ITB3; MP2K2; P85A
Plasma Membrane	ciliary basal body [GO:0036064]	MP2K1; RASN/RASH
Plasma Membrane	cilium [GO:0005929]	DLDH; PPID
Plasma Membrane	motile cilium [GO:0031514]	DLDH
Proteasome	proteasome accessory complex [GO:0022624]	PSD13
Proteasome	proteasome regulatory particle [GO:0005838]	PSD13
Synapse	cerebellar climbing fiber to Purkinje cell synapse [GO:0150053]	MGLL
Synapse	parallel fiber to Purkinje cell synapse [GO:0098688]	MGLL
Synapse	photoreceptor inner segment [GO:0001917]	ASGL1
Synapse	Schaffer collateral - CA1 synapse [GO:0098685]	PLMN
Synapse	extrinsic component of postsynaptic membrane [GO:0098890]	C1QB; C1QC

Synapse	extrinsic component of presynaptic membrane [GO:0098888]	C1QB; C1QC
Synapse	glutamatergic synapse [GO:0098978]	C1QB; C1QC; ITB3; MP2K1; PLMN; RASN/RASH
Synapse	glycinergic synapse [GO:0098690]	ITB3
Synapse	postsynapse [GO:0098794]	C1QB; C1QC
Synapse	postsynaptic density [GO:0014069]	KCC2B; MP2K1
Synapse	postsynaptic membrane [GO:0045211]	ITB3
Synapse	presynapse [GO:0098793]	MGLL
Synapse	synapse [GO:0045202]	C1QB; C1QC; CO4B; FIBG; ITB3; MGLL; SSDH
Synapse	synaptic membrane [GO:0097060]	ITB3

Supplemental Table 3. Gene Ontology Biological Process for Top 5 Mitochondrial Proteins Detected in Interaction Term.

Function	GO Biological Process	GO ID	Proteins
Mitochondrial respiration and energy metabolism	Aerobic respiration	GO:0009060	MDHM, NDUA6, NDUA8, NDUAB, NDUB2, NDUB4, NDUB8, NDUB9, NDUF6, NDUS7
	Oxidative phosphorylation	GO:0006119	COX6C
	Proton motive force-driven mitochondrial ATP synthesis	GO:0042776	ATP5E, NDUA6, NDUA8, NDUAB, NDUB2, NDUB4, NDUB8, NDUB9, NDUF6, NDUS7
	Mitochondrial electron transport, NADH to ubiquinone	GO:0006120	DLDH, NDUA8, NDUB8, NDUB9, NDUF6, NDUS7
	Respiratory electron transport chain	GO:0022904	CYC, NDUF6
	Tricarboxylic acid cycle	GO:0006099	CISY, MDHM, IDHG1, ODO2, ODPA, SUCA
	Malate–aspartate shuttle	GO:0043490	AATM, MDHM
	Malate metabolic process	GO:0006108	MDHM
	Oxaloacetate metabolic process	GO:0006107	AATM
Mitochondrial substrate transport and membrane function	Mitochondrial ADP transmembrane transport	GO:0140021	ADT1, ADT2
	Mitochondrial ATP transmembrane transport	GO:1990544	ADT1, ADT2
	Regulation of mitochondrial membrane permeability	GO:0046902	ADT1, ADT2
	Mitochondrion organization	GO:0007005	NDUF6
Mitochondrial oxidative stress	Glutathione metabolic process	GO:0006749	GSTA3, GSTA4, GSTK1
	Reactive oxygen species metabolic process	GO:0072593	NDUF6
	Hydrogen peroxide metabolic process	GO:0042743	CYC
Fatty acid and amino acid metabolism	Fatty acid beta-oxidation	GO:0006635	HCD2
	Fatty acid beta-oxidation using acyl-CoA dehydrogenase	GO:0033539	ACADS
	Fatty acid biosynthetic process (mitochondrial)	GO:0006633	MECR, NDUAB
	Branched-chain amino acid catabolic process	GO:0009083	DLDH, IVD, ODBA, ODBB
	Valine catabolic process	GO:0006574	HIBCH
	L-leucine catabolic process	GO:0006552	IVD, MCCA
	Ketone body catabolic process	GO:0046952	SCOT1
Mitochondrial biogenesis	Mitochondrial tRNA 3′-end processing	GO:1990180	HCD2
	Mitochondrial tRNA 5′-end processing	GO:0097745	HCD2
	Mitochondrial tRNA methylation	GO:0070901	HCD2
	Mitochondrion organization	GO:0007005	NDUF6

Supplemental Table 4. Muscle used for each figure.

**Figures**	**Panel**	**Muscle used**	**Effects of aging**<br>**Effects of GHSR-1a KO in aged mice**
Figure 1	E-F: muscle mass	Sol, GAS/PL, TA, EDL, Quad	Muscle mass: ↓-<br>Grip strength: ↓↑<br>Endurance: ↓↑
Figure 2	All: fiber type and size	PL	IIB fiber size: ↓-<br>IIB fiber %: ↓↑ (24m only)
Figure 3	All: muscle physiology	TA	Muscle mass: ↓-<br>Peak force: ↓-<br>Fatigue resistance (absolute force): ↓↑
Figure 4	A - OCR; B - OXPHOS; D - CS activity in isolated mitochondria	PL	Mitochondrial respiration: ↓-<br>OXPHOS CIV: ↓- (less age effects on KO)<br>Total CS activity: ↓↑
Figure 4	E, F – SDH staining	PL	SDH activity: ↓- (less age effects on KO)
Figure 4	G - mtDNA	PL	mtDNA: ↓- (less age effects on KO)
Figure 4	G, H – PGC-1α, p62 protein content	GAS	Mitochondrial biogenesis: ↓↑<br>Mitophagy: ↓↑
Figure 4	G, H - Sirt1, PINK1 gene expression	Quad	
Figure 4	J - Parkin/VDAC1 protein content in isolated mitochondria	TA	
Figure 4	J - Tomm20/LC3 colocalization	TA	
Figure 5	All: proteomics	GAS	Mitochondria are the main cellular location associated with muscle mass and function
**Figures**	**Panel**	**Muscle used**	**Effects of PF-5190457**
Figure 6	H, I: PGC-1α, p62, and LC3II protein content and Sirt1, Nrf1, Bnip3, Park2, PINK1 gene expression	GAS	Mitochondrial biogenesis and Mitophagy: ↑ in middle-aged mice
Figure 6	K: OCR in isolated mitochondria	PL	Mitochondrial respiration: no change in middle-aged mice
Sup. Fig.2	H: OCR in isolated mitochondria	PL	Mitochondrial respiration: ↑ in aged mice

Sol: Soleus

GAS: Gastrocnemius

PL: Plantaris

TA: Tibialis anterior

EDL: Extensor digitorum Longus

Quad: Quadriceps

Supplemental Table 5. Absolute values and effect sizes (with 95% CI) of weekly measures in middle-aged male mice with DMSO or PF-5190457 treatment.

	DMSO			PF-5190457		
**BW (g)**						
	Mean	Upper Limit	Lower Limit	Mean	Upper Limit	Lower Limit
Wk0	38.12	40.78	35.47	37.88	39.43	36.34
Wk1	36.19	38.47	33.91	36.52	38.11	34.92
Wk2	36.58	38.78	34.37	36.17	37.69	34.66
Wk3	36.80	39.63	33.97	35.23	36.36	34.10
Wk4	36.59	39.41	33.77	34.71	35.79	33.63
**Fat mass (g)**						
	Mean	Upper Limit	Lower Limit	Mean	Upper Limit	Lower Limit
Wk0	7.30	9.37	5.23	7.21	8.76	5.67
Wk1	6.71	8.76	4.67	7.00	8.37	5.62
Wk2	6.82	8.81	4.84	6.50	7.84	5.17
Wk3	7.57	9.61	5.53	5.66	6.77	4.55
Wk4	7.92	9.97	5.87	5.42	6.45	4.38
**LBM (g)**						
	Mean	Upper Limit	Lower Limit	Mean	Upper Limit	Lower Limit
Wk0	28.68	30.31	27.06	28.14	29.19	27.09
Wk1	27.57	29.11	26.03	27.43	28.29	26.58
Wk2	27.69	29.16	26.22	27.41	28.23	26.59
Wk3	26.77	28.34	25.21	26.90	27.81	26.00
Wk4	26.70	28.19	25.22	26.94	27.78	26.10
**Grip strength (g)**						
	Mean	Upper Limit	Lower Limit	Mean	Upper Limit	Lower Limit
Wk0	153.44	166.14	140.75	163.45	173.41	153.50
Wk1	144.00	150.85	137.15	135.09	145.38	124.80
Wk2	138.89	144.11	133.67	136.82	143.53	130.11
Wk3	145.78	157.24	134.31	146.18	157.92	134.44
Wk4	142.11	150.85	133.38	152.18	158.46	145.90
**Treadmill running time (s)**						
	Mean	Upper Limit	Lower Limit	Mean	Upper Limit	Lower Limit
Wk0	991.22	1123.63	858.81	922.00	1010.43	833.57
Wk1	930.44	1028.44	832.45	905.91	1027.79	784.03
Wk2	888.89	986.07	791.70	915.00	1046.38	783.62
Wk3	856.44	955.95	756.94	933.18	1010.97	855.40
Wk4	838.56	947.82	729.29	954.64	1044.54	864.73
**Cumulative food intake (g)**						
	Mean	Upper Limit	Lower Limit	Mean	Upper Limit	Lower Limit
Wk0	3.55	3.83	3.26	4.04	4.69	3.39
Wk1	27.59	29.16	26.02	27.95	31.18	24.71
Wk2	52.05	55.62	48.48	53.47	59.52	47.41
Wk3	77.75	82.40	73.09	78.96	86.54	71.38
Wk4	101.03	107.12	94.94	105.24	115.93	94.55

Supplemental Table 6. P-values of statistical analysis.

p-values		Main effect			WT			KO			6m	24m	28m
Two-way ANOVA		G x A	G	A	6m vs. 24m	6m vs. 28m	24m vs. 28m	6m vs. 24m	6m vs. 28m	24m vs. 28m	WT vs. KO	WT vs. KO	WT vs. KO
Figure 1	BW	0.608	0.009	<0.001	0.015	0.905	0.079	<0.001	0.389	<0.001	0.016	0.482	0.107
	LBM	0.062	0.021	<0.001	0.415	0.105	0.826	<0.001	<0.001	0.886	<0.001	0.820	0.552
	FM	0.732	0.786	<0.001	0.869	0.038	0.032	0.216	0.012	<0.001	0.554	0.640	0.636
	FI	0.646	0.134	0.858	0.673	0.917	0.939	0.974	0.688	0.898	0.837	0.290	0.221
	Hindlimb muscle mass	0.022	0.039	<0.001	<0.001	<0.001	<0.001	0.019	<0.001	<0.001	<0.001	0.966	0.852
	Sol	0.095	0.424	<0.001	<0.001	<0.001	0.108	0.067	<0.001	0.044	0.021	0.884	0.550
	GAS/PL	<0.001	0.051	<0.001	<0.001	<0.001	<0.001	0.002	<0.001	<0.001	<0.001	0.931	0.240
	TA	0.082	0.429	<0.001	0.425	<0.001	<0.001	>.999	<0.001	<0.001	0.038	0.628	0.264
	EDL	0.506	0.705	<0.001	0.062	0.003	0.699	0.675	0.001	0.022	0.331	0.498	0.582
	Quad	0.267	0.037	<0.001	<0.001	<0.001	0.067	0.039	<0.001	<0.001	0.010	0.971	0.205
	Grip strength	0.214	0.017	<0.001	<0.001	<0.001	0.892	<0.001	<0.001	0.483	0.986	0.042	0.053
	Grip/LBM	0.852	<0.001	<0.001	<0.001	<0.001	0.746	<0.001	<0.001	0.497	0.005	0.081	0.050
	Treadmill	0.059	<0.001	<0.001	0.001	<0.001	0.019	0.204	<0.001	<0.001	0.577	0.011	<0.001
Figure 2	No_IIA	0.106	0.435	0.420	0.243	0.695	0.638	0.585	0.590	0.126	0.712	0.048	0.460
	No_IIB	0.584	0.030	0.001	0.731	0.023	0.246	0.815	0.074	0.023	0.411	0.062	0.303
	No_neither	0.883	0.998	0.193	0.891	0.230	0.596	0.806	0.576	0.965	0.964	0.779	0.673
	%IIA	0.249	0.034	0.001	0.379	0.019	0.506	0.774	0.028	0.007	0.489	0.023	0.639
	%IIB	0.275	0.042	0.001	0.597	0.018	0.297	0.475	0.095	0.007	0.719	0.029	0.448
	%neither	0.864	0.472	0.682	0.836	0.580	0.953	0.906	0.977	0.960	0.502	0.612	0.986
	CSA_IIA	0.644	0.828	0.095	0.630	0.519	0.162	0.343	0.967	0.417	0.453	0.905	0.576
	CSA_IIB	0.760	0.446	0.002	0.274	0.036	0.824	0.053	0.049	0.922	0.897	0.358	0.904
	CSA_neither	0.412	0.343	0.381	0.234	0.998	0.199	0.955	0.868	0.989	0.793	0.165	0.750
Figure 3	Po	0.666	0.984	0.005		0.035			0.049		0.788		0.725
	Spo	0.617	0.811	0.629		0.524			0.989		0.632		0.837
	TA muscle mass	0.050	0.800	<0.001		<0.001			0.020		0.150		0.180
	FF_10	0.816	0.908	0.889		0.812			0.941		0.939		0.787
	FF_30	0.822	0.740	0.447		0.527			0.667		0.716		0.934
	FF_50	0.898	0.737	0.573		0.780			0.581		0.762		0.872
	FF_75	0.753	0.905	0.257		0.353			0.508		0.777		0.880
	FF_100	0.708	0.774	0.007		0.045			0.055		0.954		0.607
	FF_125	0.536	0.639	0.002		0.016			0.037		0.922		0.399
	FF_150	0.371	0.530	0.001		0.007			0.038		0.860		0.239
	FF_200	0.323	0.390	0.001		0.005			0.035		0.932		0.155
	FT_baseline	0.348	0.468	0.008		0.023			0.128		0.884		0.217
	FT_0.5	0.231	0.121	0.004		0.011			0.130		0.803		0.044
	FT_1	0.369	0.029	0.012		0.031			0.152		0.356		0.023
	FT_2	0.575	0.016	0.043		0.339			0.035		0.045		0.148
	FT_3	0.665	0.017	0.100		0.434			0.089		0.055		0.127
	FT_4	0.665	0.017	0.100		0.434			0.089		0.055		0.127
	Norm_FT_0.5	0.259	0.003	0.070		0.066			0.550		0.179		0.003

	Norm_FT_1	0.594	0.002	0.260		0.297			0.619		0.069		0.007
	Norm_FT_2	0.301	0.008	0.536		0.790			0.177		0.013		0.183
	Norm_FT_3	0.238	0.015	0.810		0.549			0.243		0.017		0.311
	Norm_FT_4	0.367	0.011	0.938		0.602			0.419		0.021		0.192
Figure 4	OCR-baseline	0.959	0.620	0.008		0.070			0.046		0.729		0.718
	OCR-ADP	0.588	0.994	0.002		0.011			0.046		0.735		0.655
	OCR-Oligo	0.528	0.195	0.029		0.054			0.243		0.669		0.120
	OCR-FCCP	0.296	0.868	<0.001		0.001			0.030		0.576		0.325
	OCR-Anti	0.798	0.462	0.015		0.062			0.103		0.760		0.420
	OXPHOS_CV	0.710	0.449	0.610		0.926			0.508		0.436		0.778
	OXPHOS_CIII	0.680	0.383	0.990		0.776			0.763		0.376		0.737
	OXPHOS_CIV	0.253	0.518	0.058		0.045			0.547		0.219		0.714
	OXPHOS_CI	0.532	0.899	0.614		0.451			0.926		0.603		0.717
	OXPHOS_CII	0.207	0.977	0.220		0.100			0.978		0.391		0.347
	CS activity	0.008	0.920	<0.001		<0.001			0.160		0.070		0.040
	SDH	0.670	0.280	0.004	0.620	0.008	0.130	0.630	0.130	0.480	0.280	0.410	0.950
	PGC-1a	<0.001	0.009	0.443	0.225	0.002	0.059	0.991	0.047	0.060	0.256	0.507	<0.001
	p62	0.002	0.591	0.371	0.072	0.088	0.994	0.008	0.419	0.107	0.012	0.008	0.466
	Sirt1*	0.601	0.067	0.416		0.225			0.916		0.049		0.728
	PINK1*	0.210	0.115	0.197		0.338			0.059		0.728		0.015
	mtDNA*	0.283	0.311	0.012		0.034			0.294		0.834		0.083
	Parkin/VDAC*	0.214	0.430	0.268		0.391			0.906		0.568		0.034
	Tomm20/LC3 (t-test)												0.042
Sup.Fig1	Free water	0.410	0.085	<0.001		<0.001			<0.001		0.499		0.087
Sup.Fig2 A (t-test)	Delta_baseline												0.820
	Delta_ADP												0.380
	Delta_Oligo												0.330
	Delta_FCCP												0.040
	Delta_Antimycin												0.670
Sup.Fig2 B	Cytochrome C	0.065	0.342	0.141		0.773			0.021		0.040		0.520
Sup.Fig3	%Collagen	0.881	0.125	0.003		0.021			0.036		0.323		0.170

p-values	Middle-aged (Figure 6)	Old (Sup.Fig. 5)
t-test (two tailed)	DMSO vs. PF	DMSO vs. PF
Cumulative FI_wk1	0.051	0.312
Cumulative FI_wk2	0.027	0.221
Cumulative FI_wk3	0.040	0.077
Cumulative FI_wk4	0.108	0.122
BW_wk1	0.517	0.700
BW_wk2	0.808	0.651
BW_wk3	0.029	0.807
BW_wk4	0.013	0.612
Lean_wk1	0.887	0.672
Lean_wk2	0.797	0.759
Lean_wk3	0.477	0.511
Lean_wk4	0.187	0.794

Fat_wk1	0.627	0.640
Fat_wk2	0.663	0.342
Fat_wk3	<0.001	0.662
Fat_wk4	<0.001	0.421
Treadmill_wk1	0.669	0.286
Treadmill_wk2	0.321	0.742
Treadmill_wk3	0.059	0.509
Treadmill_wk4	0.028	0.138
Grip_wk1	0.064	0.733
Grip_wk2	0.220	0.465
Grip_wk3	0.336	0.544
Grip_wk4	0.901	0.684
Norm_Sol	0.087	1.000
Norm_GAS/PL	0.737	0.557
Norm_TA	0.548	0.989
Norm_EDL	0.904	0.959
Norm_Quad	0.071	0.483
Norm_baseline	0.547	0.016
Norm_ADP	0.589	0.001
Norm_Oligo	0.927	0.100
Norm_FCCP	0.972	0.955
Norm_Antimycin	0.150	0.901
PGC-1a	0.014	
Sirt1	0.484	
Nrf1	0.056	
P62	0.385	
LC3II	0.016	
Bnip3	0.010	
Park2	0.448	
PINK1	0.392	
Treadmill/BW_wk0	0.457	
Treadmill/BW_wk1	0.661	
Treadmill/BW_wk2	0.706	
Treadmill/BW_wk3	0.128	
Treadmill/BW_wk4	0.081	

*Non-parametric tests were performed for pairwise comparison based on normality tests.
